# The Power of Peer Leaders: Exploring the Link between Peer Leadership Behaviors and Sustainable Work Outcomes

**DOI:** 10.3390/bs14010002

**Published:** 2023-12-19

**Authors:** Charlotte M. Edelmann, Filip Boen, Jeroen Stouten, Gert Vande Broek, Katrien Fransen

**Affiliations:** 1 Department of Movement Sciences, KU Leuven, Tervuursevest 101, P.O. Box 1500, 3001 Leuven, Belgium; filip.boen@kuleuven.be (F.B.); gert.vandebroek@kuleuven.be (G.V.B.); 2 Faculty of Psychology and Educational Sciences, KU Leuven, Dekenstraat 2, P.O. Box 3725, 3000 Leuven, Belgium; jeroen.stouten@kuleuven.be

**Keywords:** shared leadership, peer leaders, teams, leadership roles, necessary condition, employee well-being, team effectiveness, OCB, team cohesion

## Abstract

Most leadership studies primarily focus on formal leaders, often overlooking the influence of leaders within the team. While prior research has shown that peer leaders can have a beneficial impact on various team outcomes, it is yet unclear which peer leadership behaviors precisely foster a supportive and sustainable work environment. Building upon the recent identification of 10 peer leadership roles and 37 underlying functions, the current study aims to investigate the relationships between these peer leadership roles and functions and key outcomes (i.e., job satisfaction, team cohesion, team effectiveness, and OCB). A total of 31 organizational teams, comprising 182 employees from diverse sectors, participated in a quantitative survey. Employing multilevel modeling analysis, the findings demonstrated that each leadership role and nearly every function predicted at least one outcome, highlighting their significance within organizational teams. Additionally, Necessary Condition Analysis revealed that specific roles and functions were necessary for generating one or more outcomes. Finally, we found that most of the significant relationships remained consistent across teams, regardless of their size, tenure, or level of team identification. These findings refine our understanding of shared leadership and how peer leaders can create a sustainable workplace by fostering employee well-being and productivity in organizational teams.

## 1. Introduction

Employee well-being has become a more prominent subject of discussion over the past two decades and has been proposed as a vital component of social sustainability within the workplace [1,2]. Broadly speaking, social sustainability within the organizational context can be conceptualized as the social dimension involving both employees and society, which is shaped by and, reciprocally, shapes business practices, thereby influencing the future conditions and environment of the organization [3,4]. From the employees’ perspective, prioritizing their well-being contributes to sustained health and happiness in their jobs, ultimately leading to enhanced performance outcomes [5]. More specifically, a sustainable workforce effectively mitigates the costs associated with burnout, absenteeism, and personnel turnover [6]. Caring for employees’ well-being is thus equally as important as focusing solely on performance outcomes. Considering that high levels of well-being yield performance improvements and vice versa, many scholars have emphasized the need to create a ‘sustainable well-being-productivity synergy’ (e.g., [2]). This synergy implies that investing in employee well-being also benefits the organization in terms of increased work performance and that both aspects are mutually reinforcing. As such, a positive feedback loop is created that can be maintained over the long term.

Given the pivotal role that organizational behavior plays in shaping employee experiences, the achievement of this synergy is contingent upon the leaders within the organization. Traditionally, scholars have conceived of leadership as a hierarchical influence process originating from a single individual within work teams—the formal leader [7] (e.g., a manager). However, a contemporary trend is emerging where team members are assuming additional responsibilities and engaging in tasks conventionally attributed to formal leaders (e.g., [8]). This phenomenon is termed shared leadership, which implies that the primary source of influence within a team not only derives from the formal leader but also from team members. As a result, leadership can be assumed by both *formal leaders*, who hold officially recognized hierarchical positions above a team, as well as by leaders within the team (i.e., *peer leaders*) acting from the position of a team member. Specifically, the conceptual framework of shared leadership, as articulated by Pearce and Conger [9] (p. 1), encapsulates “a dynamic, interactive influence process among individuals in groups for which the objective is to lead one another to the achievement of group or organizational goals or both”.

Nonetheless, the efficacy of shared leadership may hinge on the content of the leadership being shared. To attain a sustainable well-being-productivity synergy and promote enduring well-being within the workplace, it is crucial to understand how organizational behavior is related to different desired outcomes. A considerable body of research has examined the organizational behavior of formal leaders in agile or self-managing teams (e.g., [10,11]). However, less is known about the specific behaviors exhibited by peer leaders in shared leadership contexts and their contributions to desired work outcomes. Moreover, it remains unclear whether these contributions vary across different team characteristics. This knowledge gap may result in suboptimal implementations of shared leadership in practice, consuming the time and effort of both the formal leader and the team, with potentially negative consequences for the team and the organization as a whole.

Edelmann et al. [12] initiated the identification of various leadership behaviors of peer leaders. Through an extensive qualitative study encompassing interviews and expert panels, the researchers developed a peer leadership taxonomy that comprises 10 peer leadership roles and 37 functions (i.e., specific behaviors associated with each role). In the practical implementation of shared leadership, employing such a comprehensive set of roles establishes a framework that aids team members in navigating diverse behaviors. However, it remains unclear which of these identified roles and functions play a crucial role in contributing to distinct work outcomes.

Hence, to support the implementation of shared leadership, our study aims to explore the extent to which these different peer leadership behaviors contribute to creating a supportive and sustainable work environment. Specifically, our first aim is to investigate the links between the diverse leadership roles and functions as defined by Edelmann et al. [12] and key outcomes, including job satisfaction, team cohesion, team effectiveness, and OCB. We anticipate variability in these relationships, with some roles holding greater importance and/or necessity for specific outcomes than others. Secondly, we explore the potential moderating impact of team-specific characteristics. Specifically, we hypothesize that the relationships are stronger in teams with fewer members, shorter tenure, and higher levels of team identification. Our research findings largely confirm our hypotheses, demonstrating variations across leadership roles and functions. As such, the present study serves not only to enrich the discourse on shared leadership and social sustainability but also to furnish pragmatic insights for formal leaders aspiring to share the lead with their team.

### 1.1. The Benefits of Shared Leadership

Recent empirical studies and meta-analytic reviews have consistently unveiled positive associations between shared leadership structures and critical organizational outcomes. These outcomes encompass a spectrum of dimensions, including team performance and team effectiveness (e.g., [7,13]), as well as individual performance indicators like organizational citizenship behaviors (OCBs) (e.g., [14]). Furthermore, researchers have adeptly demonstrated links between shared leadership and employees’ well-being (e.g., satisfaction with one’s job or team) (e.g., [13,15]), along with team cohesion (e.g., [16]). Notably, research has demonstrated that shared leadership goes beyond the traditional realm of vertical leadership, making a significant contribution to team performance and overall effectiveness [17]. Given that shared leadership can also make a substantial contribution to overall employee well-being, it is argued that this leadership approach concurrently advances social sustainability within the workplace [18]. For instance, according to Pearce and Akanno [19], adopting a shared leadership structure is likely to yield a more resilient management system that is better equipped to facilitate organizational sustainability.

Interestingly, existing research has presented instances of null effects or even inverse correlations between shared leadership and team performance (e.g., [20,21]). Within the context of shared leadership theorization, it is necessary to elucidate these discrepancies. One plausible explanation for these divergent findings resides in the multifaceted ways by which shared leadership can be implemented, thereby influencing the resulting outcomes. Specifically, shared leadership entails team members assuming distinct roles and shouldering corresponding responsibilities within these roles. These roles align with established conceptions of effective teams in which specific roles and associated accountability are argued to be important elements for optimal team functioning (e.g., [22]). In delving deeper into these roles, the efficacy of shared leadership appears to be intrinsically linked to the content of these designated roles.

Over the past fifty years, much of the leadership research has aimed to identify the leader behaviors contributing to team performance, resulting in a plethora of studies with substantial variations in the number and types of behaviors investigated. Yukl et al. [23] noted that many of these studies concentrated on one or two broadly defined behavioral categories, with only a few delving into multiple specific leader behaviors, leading to an incomplete understanding of the diverse ways leaders contribute to team success [24]. Furthermore, Yukl et al. [23] observed that most analyses relied solely on the aggregate of the broadly defined behavioral categories without considering their underlying functions, which challenges the interpretation of leader behavior effectiveness. Thus, to advance the leadership effectiveness research, Yukl et al. [23] underscore the importance of examining the influence of the individual underlying behaviors, especially when they differ in their relevance for achieving an outcome.

Recognizing the conceptual ambiguity in the literature, numerous efforts have been made to develop more precise leadership taxonomies. For instance, a recent exploratory study by Petermann and Zacher [10] sought to inductively construct a behavioral taxonomy related to workforce agility. Nevertheless, organizational research to date has mainly centered on the distinct leadership behaviors expected from formal leaders and their consequences [7], leaving the impact of leadership behaviors fulfilled by *peer leaders* largely unexplored. As a consequence, researchers and practitioners in the field of shared leadership still encounter challenges in understanding the specific peer leadership behaviors that are important for achieving favorable outcomes [25]. This knowledge deficit may lead to suboptimal implementations of shared leadership in practice, incurring an expenditure of time and effort from both the formal leader and the team. Such implementations might not yield the anticipated positive outcomes and, in some instances, might even result in less desirable outcomes. Consequently, further research is required to delve into the pivotal roles that peer leaders can assume to advance social sustainability.

### 1.2. Functional Approach to Leadership: What Does Effective Peer Leadership Entail?

Addressing the existing ambiguity in the shared leadership literature, our research seeks to comprehensively examine the relationship between diverse leadership behaviors exhibited by peer leaders and sustainable work outcomes. To comprehend the factors contributing to effective team leadership, researchers often adopt a functional approach (e.g., [26]). In this perspective, the leader’s primary responsibility is to address whatever is not adequately managed for the group’s needs [27]. Essentially, this approach focuses on identifying team needs and determining the specific behaviors leaders should exhibit to meet those needs. In this context, the term *function* denotes any specific behavior aimed at enhancing outcomes. Leaders can be deemed effective to the extent that they ensure the performance of a defined set of leadership functions necessary to meet the team’s needs [28]. Within this framework, leadership functions that share thematic content can be combined into an overarching *leadership role* that peer leaders can occupy. According to the organizational role theory, a role constitutes the sum of behaviors expected from an individual [29,30]. Consequently, a role encompasses a cluster of content-related functions representing specific behaviors that an individual is expected to fulfill [31]. Working with overarching roles may enhance manageability, particularly for individual team members assuming leadership roles, and role clarity for everyone involved. For example, more specific functions entail a clearer definition of responsibilities, potentially reducing the likelihood of conflicts among peer leaders [32]. These roles should, however, not be used independently of their underlying functions, as these concrete functions provide a structured basis for assigning concrete responsibilities to (peer) leaders.

Over the years, numerous taxonomies have emerged to outline team roles that contribute to effective team performance. However, much of this work has been conducted on ad hoc teams functioning for short durations under low-stress conditions [33]. In addition, according to Driskell et al. [34], there is a considerable divergence in existing team role taxonomies, with recent taxonomies by Mathieu et al. [35] and Driskell et al. [34] incorporating a more varied set of roles. Despite the extensive literature on (formal) leadership and team roles, there is limited knowledge regarding the impact of *peer leadership behaviors*. Particularly, leadership is a critical element, as in shared leadership, team members are involved in influence processes directed toward collective goals. These roles specifically encompass leadership behaviors by team members. This distinguishes them from regular team roles that are typically related to specific tasks or positions in the team and that do not inherently assume goal-directed influence.

In search of more differentiated leadership behaviors for both formal leaders and peer leaders, Morgeson et al. [24] conducted a review of the existing literature and formulated a framework comprising 15 essential leadership functions. However, one limitation of this framework is that it focuses exclusively on team performance (i.e., what the team needs for goal accomplishment). We posit that functions can yield multiple beneficial outcomes, particularly in promoting social sustainability, wherein the consideration of employee well-being becomes imperative. Another limitation is that Morgeson et al.’s framework lacks differentiation between functions for formal leaders and peer leaders. This abstraction is needed and important, as peer leaders and formal leaders may exhibit different functions with potentially different outcomes.

Addressing this research gap, Edelmann et al. [36] demonstrated the benefits of distinct peer leadership roles based on four peer leadership roles that were previously identified in the sports context [37]. Given the unique characteristics of organizational settings, and in response to the aforementioned limitations, Edelmann et al. [12] conducted a comprehensive qualitative analysis involving interviews and expert panels to unravel the distinct peer leadership roles in organizational teams. Here, the researchers identified the precise behaviors (i.e., leadership functions) deemed vital by employees for sustainable performance and well-being/relationship-oriented outcomes. These leadership functions were subsequently classified into a set of distinct leadership roles, yielding a total of 10 exclusive leadership roles designated for peer leaders [12] (see Table 1 for an overview of these roles and their corresponding definitions). These roles encompass diverse aspects that have the potential to cultivate a supportive and sustainable work environment conducive to both well-being (e.g., the role of the *Connecting leader* who resolves tensions between colleagues) as well as productivity (e.g., the role of the *Task leader* who follows up on the teams’ work schedule).

However, so far, the distinct and varying contributions of these roles and functions concerning different outcomes have remained unexplored. Moreover, we posit that the implementation of shared leadership might not yield uniform results across diverse teams. Indeed, not all roles and functions may be equally important within every organizational context. It is thus necessary to consider the wide diversity of teams (e.g., in terms of team size) to formulate nuanced statements about the relevance of each role within a specific team context. We will assess the differential impact of leadership content within teams and explore whether this influence diverges across different teams. In this way, both leadership scholars and practitioners can gain a more detailed understanding of the salient peer leadership roles important for achieving specific desired outcomes. Following scholars’ advice to adopt a function-based approach when studying the content of shared leadership [7], the present study will advance the existing shared leadership literature by targeting two aims.

**Aim 1: The importance of different peer leadership roles and functions.** As previously observed by Morgeson et al. [24], earlier research has predominantly focused on the leadership behavior of formal leaders. Thus, our primary objective is to pinpoint the pivotal behaviors that *peer leaders* can undertake. We will evaluate the impact of both comprehensive roles and concrete functions, considering their previously mentioned respective benefits in the implementation of shared leadership. In doing so, we expand our knowledge of how peer leadership can promote sustainable work outcomes.

To accomplish this aim, we commenced with the peer leadership roles delineated by Edelmann et al. [12] and investigated which of the 10 specified roles is most strongly related to organizational outcomes. To respond to the call of Manheim [38] (p. 51) to “examine outcomes of shared leadership that are different from team performance and team effectiveness”, we incorporated a diverse array of organizational outcomes. Given that a sustainable well-being–productivity synergy requires leadership behaviors that prioritize both employee well-being and performance, these outcomes encompassed two well-being/relationship-oriented measures, again, one at the collective level (i.e., team cohesion) and the other at the individual level (i.e., job satisfaction), and two performance-related measures, one centered on the team (i.e., team effectiveness) and the other on the individual (i.e., Organizational Citizenship Behavior, OCB). Prior research has already demonstrated a positive link between each of these four measures and enhanced levels of employee well-being ([39,40,41,42], respectively). Due to their established link with employees’ psychological, social, and physical well-being, these measures can thereby also contribute to an organization’s social sustainability.

**Figure 1:**
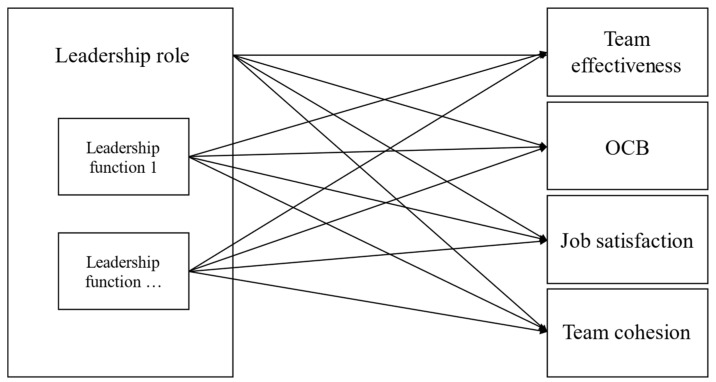
Generalized conceptual model representing the hypothesized positive relationships between each of the 10 peer leadership roles, including their corresponding leadership functions as defined by Edelmann et al. [12], and the four examined outcome variables (i.e., team effectiveness, OCB, job satisfaction, and team cohesion), as described in Aim 1.

We anticipated variations in the relationships of the 10 distinct leadership roles with the respective outcomes. Yet, which of these roles would be stronger or weaker related to each of the outcomes cannot be clearly identified from prior studies. Consequently, no clearly outlined hypotheses were postulated a priori. In addition, we extended our analytical pursuits to the level of functions that underlie each role. More specifically, within the subset of roles that are significantly linked to a particular outcome, we investigated the specific functions nested within these roles, thereby discerning the most relevant functions needed to attain that outcome. For a generalized conceptual model of these expected relationships, we refer to Figure 1.

In addition to traditional statistical methodologies such as regression analysis, we used a pioneering technique, namely Necessary Condition Analysis (NCA) [43], as an innovative facet of the scientific study of shared leadership. Using NCA, it can be determined whether a particular leadership role is a necessary (but not sufficient) condition for a specific outcome to emerge, thus functioning as a prerequisite for the achievement of that outcome. The inclusion of this supplementary analysis enriched our investigation, as it led to a more nuanced understanding of the interplay between peer leadership and the designated measured outcomes.

**Aim 2: The moderating role of team-specific characteristics.** While the exploration of the aforementioned relations is intriguing in itself, the strength of the potential effects of the 10 peer leadership roles on the four outcomes might be contingent upon distinct team characteristics. Several scholars have indeed highlighted that various team-related variables can moderate the shared leadership–outcomes relation (e.g., [9]). These variables encompass the discernible demographic composition (e.g., team size and team tenure) along with less observable cognitive states within the team (e.g., member’s identification with their team) [8,44].

***Team size*.** The influence of team size on social dynamics within groups has been repeatedly demonstrated (e.g., [45]), prompting scholars within the shared leadership field to advocate for the inclusion of team size as a variable of interest rather than merely a covariate to be statistically controlled for [25]. Notably, team size is argued to be a moderating factor that can have both advantageous and adverse impacts on teams [8].

On the one hand, researchers posit that cultivating close collaborative bonds within large teams presents a challenge, potentially hindering the emergence and establishment of shared leadership structures (e.g., [46]). Consistent with this idea, Edelmann et al. [36] observed that the relation between the perceived quality of peer leadership and both team effectiveness and job satisfaction was stronger within smaller teams compared to larger teams. One possible explanation for this finding is that as team size increases, proximity barriers among team members emerge, resulting in a more ambiguous sense of team objectives, reduced participation, and less optimal team coordination [8,47]. On the other hand, scholars have argued that larger teams might harbor a greater capability for decision making and information processing, facilitating the reciprocal process of influence among team members (i.e., the essence of shared leadership) [8]. Finally, contrary to the aforementioned perspectives, a number of studies did not yield a moderating role of team size. For instance, team size was not found to moderate the relation between shared leadership and team effectiveness [48] nor the relation with burnout [36].

Therefore, we hypothesized that team size moderates the relation between peer leadership across the diverse roles and the different outcomes (H1). However, the divergent empirical findings concerning the nature of its moderating effect warrant further exploration. Given that most researchers provide arguments for a stronger effect of shared leadership in smaller (rather than larger) team settings, we expected a similar trend here as well—that is, the relations were anticipated to be stronger in teams that count fewer members.

***Team tenure.*** Scholars have postulated that shared leadership may harm team performance in teams that work together for a long period [8]. For instance, Wu and Cormican [49] observed the evolution of shared leadership across different project phases, which led them to propose that “the optimal level of shared leadership appears in the early phase of a project” (p. 299). This phenomenon can potentially be attributed to the intricate nature of power distribution inherent in shared leadership, which proves challenging to consistently manage and balance throughout time [50]. This, in turn, gives rise to a greater risk of power struggles and tensions in teams with a longer tenure, which can disrupt overall team performance. In light of these considerations, we hypothesized that team tenure moderates the relation between the distinct peer leadership roles and the different outcomes (H2) so that this relation is stronger in teams with shorter (as opposed to longer) tenure.

**Figure 2:**
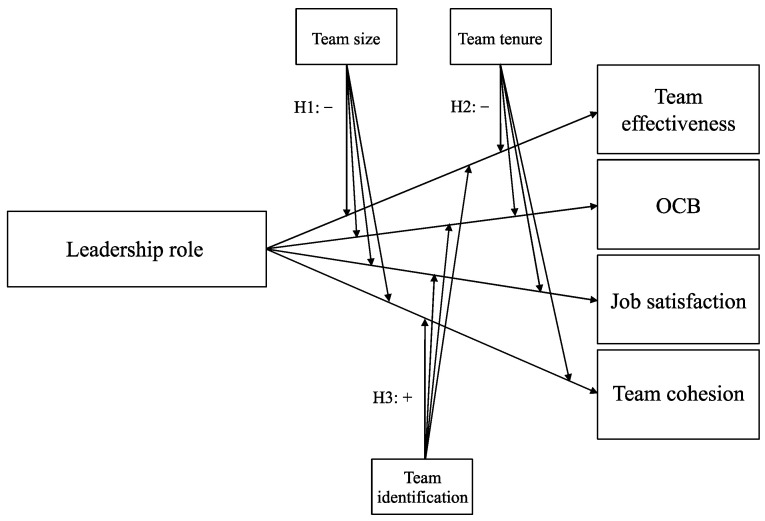
Generalized conceptual model representing the hypothesized moderating effects of team size, team tenure, and team identification on the relationship between each leadership role (i.e., as the average of its underlying functions) as defined by Edelmann et al. [12] and each of the four examined outcome variables.

***Team identification.*** Team identification is the extent to which team members think and behave in terms of their social identity (termed “us”), transcending their personal identity (“you” or “me”). Prior organizational research has evidenced its positive impact on team effectiveness and team satisfaction (e.g., [51]). Drawing upon this foundation, scholars in the field of shared leadership argue that the absence of a shared collective identity could impede the effectiveness of shared leadership structures [7]. Shared leadership may thus be more (or only) effective when a social identity is collectively embraced, creating a unified orientation. We, therefore, hypothesized that team identification operates as a moderating factor in the relation between the diverse peer leadership roles and the four measured outcomes (H3), so that this relation would be stronger in teams in which team identification was relatively high (rather than low). Figure 2 illustrates the expected moderating effects as described in H1–H3.

## 2. Method

### 2.1. Data Collection

**Procedure.** A cross-sectional quantitative research design was employed to achieve the two research aims. We invited individual employees working within a team context (i.e., either as a team member or as the formal leader of that team) to participate in this study via email through our professional and personal network. In doing so, a purposive sampling approach was followed, adhering to specific inclusion criteria. Eligible participants were required to be a minimum of 18 years of age, employed in Belgium, proficient in Dutch, and either affiliated with a team helmed by a direct formal leader or assuming the role of a formal leader themselves. Those who were self-employed or functioned within self-managing teams were consequently precluded from participation. This deliberate exclusion served to differentiate teams led by designated formal leaders from self-managing teams, anticipating potential variations in leadership behaviors. By doing so, we aimed to avoid confusion and maintain sample homogeneity, thereby upholding the internal validity of our findings and leaving open the possibility for future research to compare results across different team structures. In addition, we based ourselves on the team definition of Kozlowski and Ilgen [52], prompting us to exclusively select teams that comprised a minimum of six team members, including the formal leader, who engaged in reciprocal interactions (be it virtually or face to face).

In tandem with these selection criteria, our recruitment approach adhered to a predetermined stratification framework aimed at securing a sample encompassing the various layers of society and reflecting the diversity of organizational teams in Belgium. To be specific, the sample was stratified across sectors (profit vs. non-profit) and the hierarchy level in which participants were situated in their organization, distinguishing between higher levels (i.e., the upper management and executive level) and lower subordinate levels (see Appendix A for an overview).

Ethical approval to conduct this study was obtained from the Ethics Committee at KU Leuven (G-2020-1658). Initially, participants received an email containing an online link directing them to the survey, which they completed using either a computer or a mobile phone. Before participating, individual participants within the same team were assigned unique team codes, which were communicated to them through the same email. These codes enabled the aggregation of responses on a team basis, facilitating subsequent team-level analyses. At the outset of the survey, participants were instructed to enter their team code and to provide informed consent, wherein their anonymity and confidentiality were guaranteed. Participation in the study was voluntary and not reimbursed.

**Participants.** A power analysis using the G*Power 3 software revealed that for the intended analyses a total of 138 participants was needed to detect a medium effect size of 0.30, based on Chen et al. [53], with a significance level of 0.05 and a power of 0.95. Given the stipulation that each team must comprise a minimum of six participants, the objective was to enlist no fewer than 26 teams. Recruitment of these teams occurred in two stages. In the first stage, 131 teams were approached, out of which 29 teams fulfilled the inclusion criteria and participated in the study (i.e., a response rate of 22.14% mainly due to time constraints). In the second stage, we recruited seven additional teams to ensure the attainment of a satisfactory sample size following our predetermined stratification scheme. In total, 194 respondents, nested in 36 teams, completed the survey.

In the next step, we undertook a two-fold approach to ascertain the reliability and validity of the collected data (e.g., consistency of responses within a team). First, three attention-check questions were strategically incorporated within the survey, necessitating participants to deliberately choose specific response options. By this means, we were able to gauge the conscientiousness with which participants engaged in the survey completion process. Data was retained solely from participants who correctly addressed all attention checks, ensuring data integrity for subsequent analysis. Second, only the teams in which, aside from the formal leader, at least three (of the required minimum of five) team members completed the survey were included in the final sample. This decision was guided by prior research (e.g., [54,55]), which exclusively involved teams with a minimum of three members completing the survey, aligning with the requirements for the multilevel model method (i.e., at least 30 teams and at least three respondents per team) [56].

The final sample encompassed 182 respondents nested in 31 distinct teams, thereby satisfying all requirements for the execution of multilevel analyses [56]. More specifically, for data analysis, we used the responses obtained from 31 formal leaders and 151 team members, as detailed in Appendix B. Within this conclusive sample, the mean count of respondents per team was six individuals, including one formal leader (*M* = 5.87, *SD* = 1.41). Per work week, participants reported engaging in face-to-face interaction with at least one team member for an average of 17.13 h (*SD* = 12.95) and digital interaction for an average of 10.40 h (*SD* = 34.60). Overall, participants indicated that the interaction with their team comprised both in-person (50.15%) and digital (49.85%) communication, presumably due to the prevalent COVID-19 restrictions during the time of data collection.

The participants worked in organizations operating in either the profit sector (*n* = 14) or the non-profit sector (*n* = 17). These micro, small, medium-sized, and large organizations were located in Belgium and were representative of a diverse range of industries, including healthcare, agriculture, ICT, justice, and safety. The overall team size, as indicated by the participating team members, ranged from 5 to 28 members, excluding the formal leader (*M* = 11.76, *SD* = 5.68). Additionally, the participants’ team tenure spanned a range of 2 to 12 years. Further information concerning the demographics of the participating formal leaders and team members can be found in Table 2.

### 2.2. Measures

The study involved the administration of two distinct surveys, one for the team members and another for their respective formal leaders. All measures were based on self-reported data. Their constituent items originally articulated in English were translated into Dutch and then assessed using the back-translation method performed by different individuals to ensure content validity across the languages [57].

**Exhibition of peer leadership functions in the team.** Following the provision of demographic information, both team members and their respective formal leader were prompted to evaluate the manifestation of each of the 37 peer leadership functions (displayed in Table 3) among their fellow team members, whom they considered to hold a peer leadership role within their team, on a scale ranging from 0 (*not at all*; indicating no manifestation) to 10 (*very much*; indicating a substantial manifestation). The researchers of this study previously developed and formulated the content for each leadership function through an extensive qualitative study approach (see [12] for more comprehensive details about the development of these functions). The choice to employ these functions as measurements, rather than aggregating them and using roles as the primary measurement, was based on the rationale presented by Yukl et al. [23]. They argued that examining the underlying components within a broader role is essential for understanding how specific functions within a role vary in their importance for a given outcome, thereby enabling a more accurate interpretation of leader effectiveness. Throughout the survey, the description of peer leadership was presented as follows: “In addition to (mention the formal leader’s name) as your formal leader, it is common to observe ‘leaders’ emerging *within* your team. These are one or more colleagues without a formal leadership title, but they informally exhibit leadership, thereby enhancing the team’s functioning and/or the well-being of its members”.

Given that the initiation of data collection coincided with the onset of the COVID-19 crisis, potentially changing participants’ work situations, we advised participants to reflect upon their colleagues *before* the outbreak when rating the peer leadership functions. Moreover, participants were explicitly instructed to select the response option “not applicable” for leadership functions that did not apply to their situation because their work environment did not facilitate the demonstration of a particular behavior.

**Outcome measures.** Both team members and formal leaders provided ratings on scales measuring performance and well-being/relationship-oriented outcomes to secure diverse data sources and potentially reduce common method variance bias [58]. Specifically, the perceived team effectiveness (26 items with seven subscales; e.g., “The team is highly effective at implementing solutions“) [17] and organizational citizenship behavior of team members (OCB; 16 items with two subscales directed towards other individuals and the organization; e.g., “I offer ideas to improve the functioning of our team“) [59] were rated on a 7-point Likert scale spanning from 1 (*completely disagree; never*) to 7 (*completely agree; always*). Perceived job satisfaction (three items of the Job Diagnostic Survey by Hackman and Oldham [60]; e.g., “Generally speaking, I am very satisfied in this team“.) was appraised on a 7-point Likert scale ranging from 1 (*completely disagree*) to 7 (*completely agree*).

Additionally, the constructs of team cohesion and team identification were exclusively evaluated by team members, a choice grounded in the expectation that these individuals would likely provide more accurate assessments of these variables compared to formal leaders. Team cohesion (six items with two subscales, interpersonal-oriented and task-oriented; e.g., “There is a feeling of unity and cohesion in my team“) [61] and team identification (using the single-item social identification measure by Postmes et al. [62]; e.g., “I identify with this team“) were measured on a 7-point Likert scale ranging between 1 (*completely disagree)* and 7 (*completely agree*).

For capturing team tenure, we computed the mean duration of engagement reported by both individual team members and the formal leader within their respective current teams and then assigned this mean value to each participant belonging to the same team. In doing so, we could compare potential differences between newly constituted teams and more mature teams. Similarly, we aggregated the individual scores on team identification with each team and extended this resultant average score to the whole team. This aggregation aimed to facilitate an examination of the degree to which a team shared a sense of team identification and whether this collective sense of ‘us’ holds positive implications for outcomes compared with teams with a lower average level of team identification.

### 2.3. Data Analysis

To address Aim 1, we conducted a regression analysis to assess the contributions of various roles and their respective functions toward distinct outcomes (i.e., team effectiveness, OCB, job satisfaction, and team cohesion). Given that Aim 1 of the current study was to assess the individual predictive value of each role and function, a simple multilevel regression analysis was employed as the appropriate method for examining distinct models, as opposed to multiple multilevel regression analysis, which would be more suitable for addressing different research questions. To achieve this, we employed a multilevel regression modeling technique in R software (v. 4.3.2) [63], which enabled us to account for the nested data in our sample (i.e., participants grouped within teams). It is contended that expanding shared leadership research to include a multilevel analysis is essential for a comprehensive understanding of shared leadership theory [64]. As a result, a series of nested regression models were constructed, wherein a specific peer leadership role was treated as a between-subject variable, and a specific work outcome served as the dependent variable. A random intercept was added, allowing us to infer relations unaffected by the clustered nature of our data, which could potentially inflate standard errors, thus allowing the capture of variations solely between individuals [65]. All predictors in the regression models were grand mean-centered to enhance the interpretability of our findings. The same approach was adopted to examine potential differences between profit and non-profit organizations, as well as variations between high and low levels of perceived power distribution within teams (see Appendix C and Appendix D for the results).

Additionally, we explored for each outcome which leadership roles were indispensable (i.e., a necessary condition) for the realization of an outcome, rather than merely being sufficient conditions. In this context, a role qualifies as necessary if the prediction of outcomes would not hold statistical significance in its absence. The necessary conditions within our dataset were investigated using Necessary Condition Analysis (NCA) as conceptualized by Dul [43]. Rooted in necessity logic, this analysis identifies the necessary but not sufficient contributions made by predictors (e.g., a specific leadership role) towards outcomes (e.g., team effectiveness). Operating on the premise of multiplicative causality (i.e., Y = a · b1X1 · b2X2 · b3X3 · …), the outcome attains a value of zero as soon as any predictor assumes the value of zero. Put simply, without this predictor, the desired outcome cannot exist (i.e., it is necessary). A common example to illustrate this logic is that the presence of air to breathe is a necessary condition for human life. Still, air by itself is not sufficient to sustain life, given that human existence also requires other necessary conditions (e.g., water, food, safe environment). While NCA is a relatively recent method, it is increasingly utilized or recommended in various business and management disciplines [66]. Indeed, over recent years, it has been applied in numerous leadership studies (e.g., [67]) and within the context of employee well-being (e.g., [68]). Following Dul et al.’s [66] suggestion to use NCA in conjunction with other methods, we initially identified the factors contributing to the outcome through regression analyses before assessing if these factors were also essential (i.e., necessary). Thus, after testing the collective contribution of the 37 leadership functions inherent in a leadership role through traditional multilevel regression analysis, we subsequently turned to NCA at the level of the functions. While neither approach is superior to the other, it is important to conceptually distinguish between the traditional and necessity approaches, as they are often confused by researchers [69].

We used the NCA package [70] in R to examine which roles are indispensable for yielding benefits and thus must unequivocally be fulfilled in teams embracing a shared leadership structure with a targeted outcome. At a deeper level, we can also examine which functions (i.e., positive leadership behaviors) within a given leadership role are particularly necessary (i.e., need to be exhibited) to achieve the outcome. By focusing on these necessary leadership functions, peer leaders will be able to fill out their leadership roles most efficiently. The presence of a necessary condition is visually apparent through an empty zone above the ceiling line within a scatter plot. The larger this empty zone, the stronger the effect [43]. Following the benchmark of Dul [43], the effect size of a necessary condition (*d*) can be classified as small (0 < *d* < 0.10), medium (0.10 ≤ *d* < 0.30), large (0.30 ≤ *d* < 0.50), or very large (*d* ≥ 0.50). Given the discrete nature of our study variables with a constrained range of levels, we used the ceiling envelopment–free disposal hull ceiling technique (CE-FDH) and 10,000 permutations to compute significance levels [71].

To address Aim 2, we investigated the potential moderating role of team characteristics in the relation between peer leadership roles and the four outcome variables. In preparation for data analysis, the outliers for each moderator were identified and removed from the dataset: four teams (comprising 26 data points) that reported a team size of 35 or more team members, and four outliers of individual responses for team tenure. We realize that the number of teams might not reach a level high enough to yield robust interaction effects. Nevertheless, we firmly believe that performing these moderation analyses generates valuable insights into the underpinning mechanisms. We then incorporated a two-way interaction term with a peer leadership role and team size as an additional predictor within the 10 multilevel regression models employed in the main analyses. In the case of a significant interaction effect, a simple slope analysis [72] was performed to unravel the nature of the significant interaction. Here, we tested the separate effects of the higher levels (1 *SD* above the mean) versus the lower levels (1 *SD* below the mean) of a given moderator variable (e.g., a greater vs. smaller number of team members). An analogous procedure was implemented for the remaining two moderator variables: team tenure and team identification.

## 3. Results

### 3.1. Aim 1: The Importance of Different Peer Leadership Roles and Functions

Table 3 provides an overview of the (inter)correlations among all predictors (i.e., roles and functions) and the four outcome measures, including their means, standard deviations, and Cronbach’s alpha values, representing the internal consistency of each measure. The values for the predictor measures (i.e., the leadership roles) ranged between 0.60 and 0.84, indicating a moderate to good level of reliability. All values for the outcome measures exceeded 0.80, and thus the reliability of our data was ensured [73]. The intercorrelations between the different roles were all significant (all *p*’s < 0.01), except for the correlation between *Social activity leadership* and *Logistics leadership* (where *p* = 0.015), and ranged from 0.23 to 0.81. The correlation patterns between the peer leadership roles and the different outcomes were consistent with our hypothesized relations. Interestingly, all 10 roles showed (small to moderate) significant relations with both team effectiveness and OCB, with some roles also displaying significant correlations with job satisfaction and team cohesion. It is important to note that both formal leaders and team members completed the survey. We did, however, conduct additional analyses to scrutinize potential variations in the observed relationships when including versus excluding the formal leader. The findings remained highly consistent even when formal leaders were omitted from the sample, although a comprehensive analysis of differences may be constrained due to the limited size of our formal leader sample.

Concerning team cohesion, we also examined its two dimensions (i.e., task-oriented and interpersonal-oriented team cohesion) separately. Besides some similarities (i.e., the roles of *Connecting*, *Social activity*, *Motivational*, and *Unity leadership* were related to both dimensions), the roles of *Task*, *Critical Innovation*, and *External leadership* were found to solely relate to task-oriented team cohesion (i.e., *r* = 0.21, *p* = 0.026; *r* = 0.21, *p* = 0.020; and *r* = 0.19, *p* = 0.046, respectively). Conversely, the *Exemplary leadership* role was exclusively linked to interpersonal-oriented team cohesion (i.e., *r* = 0.19, *p* = 0.030). While not all roles correlated with job satisfaction and team cohesion, it is crucial to acknowledge that within each role, there were distinct functions (i.e., behaviors) that exhibited significant correlations. Indeed, every leadership function was significantly related to at least one outcome in the expected direction (with a small to moderate effect size).

Before embarking on the regression analyses, we computed the intraclass correlation (ICC) for each measurement. The resulting percentages representing the variance at the team level for each measure are detailed in Table 3. The results of the regression analyses for the diverse roles and their underlying functions (i.e., respective standardized parameter estimates and *p*-values) are presented in Table 4. Table 5 depicts the statistical results of the supplementary NCA (i.e., estimated effect sizes and corresponding *p*-values that indicate their significance). In addition, bottleneck tables were computed to assess the minimum required level of the necessary condition to achieve different outcome levels (see Appendix E).

**Table 3:** Means, standard deviations, reliability, intraclass correlation coefficients, and significant correlations between the 10 peer leadership roles and the underlying 37 peer leadership functions defined by Edelmann et al. [12] on the one hand and all included scales of outcome measures on the other hand.

Leadership Roles and Their Underlying Functions (Defined by Edelmann et al. [12])	*n*	*M*	*SD*	Team Effectiveness (*α* = 0.96; ICC = 0.28)	OCB (*α* = 0.89; ICC = 0.30)	Job Satisfaction (*α* = 0.84; ICC = 0.15)	Team Cohesion (*α* = 0.94; ICC = 0.30)
**Task leader** (*α* = 0.77; ICC = 0.12)	-	7.00	1.53	0.34 **	0.35 **	0.13	0.18
Ensuring a fair division of tasks	161	6.81	2.30	0.27 **	0.25 **	0.10	0.15
Monitoring the work	166	7.38	1.90	0.18 **	0.22 **	0.24 **	0.08
Representing the team internally	158	7.12	1.96	0.30 **	0.34 **	0.08	0.12
Creating clarity about each other’s work	164	6.99	1.96	0.33 **	0.34 **	0.19 *	0.27 **
Coordinating tasks at team meetings	155	6.41	2.66	0.10	0.16 *	−0.05	0.11
**Connecting leader** (*α* = 0.82; ICC = 0.07)	-	6.83	1.63	0.24 **	0.33 **	0.13	0.24 **
Being a confidential advisor for colleagues	173	7.14	2.02	0.19 *	0.23 **	0.13	0.12
Cultivating a healthy work culture	170	6.96	2.28	0.20 **	0.24 **	0.07	0.11
Reducing tensions within the team	167	6.32	2.45	0.12	0.21 **	0.10	0.12
Opening up to colleagues	171	6.89	2.34	0.15	0.19 *	0.07	0.15
Ensuring connection and integration within the team	157	6.90	1.90	0.19 *	0.29 **	0.11	0.18 *
**Social activity leader** (*α* = 0.69; ICC = 0.27)	-	6.37	1.99	0.24 **	0.38 **	0.08	0.24 **
Cultivating a good team atmosphere at work through social activities during work hours	170	6.97	2.19	0.20 *	0.25 **	0.17 *	0.11
Organizing social activities outside work hours	161	5.27	2.99	0.14	0.33 **	−0.00	0.20 *
Paying attention to special events of team members	168	6.76	2.49	0.19 *	0.27 **	0.03	0.18 *
**Motivational leader** (*α* = 0.84; ICC = 0.06)	-	7.39	1.48	0.32 **	0.28 **	0.20 *	0.19 *
Motivating colleagues to work	173	6.79	1.93	0.25 **	0.20 **	0.16 *	0.11
Encouraging colleagues to take initiative and voice their opinion	172	7.54	1.75	0.29 **	0.22 **	0.18 *	0.15
Recognizing the work of colleagues	172	7.68	1.69	0.30 **	0.27 **	0.16 *	0.23 **
Expressing gratitude	171	7.53	1.85	0.22 **	0.23 **	0.15	0.14
**Critical innovation leader** (*α* = 0.68; ICC = 0.13)	-	7.08	1.48	0.32 **	0.37 **	0.13	0.15
Daring to take a critical look and initiating change	170	7.14	2.09	0.33 *	0.36 **	0.15	0.17 *
Daring to take initiative	173	7.39	2.05	0.13	0.16 *	0.07	0.14
Daring to criticize the formal leader	167	6.65	2.10	0.09	0.20 *	−0.03	0.01
Preparing for the future	171	7.13	2.11	0.25 **	0.28 **	0.15 *	0.08
**Team-development leader** (*α* = 0.75; ICC = 0.08)	-	7.14	1.52	0.24 **	0.29 **	0.19 *	0.17
Developing the growth and expertise of colleagues	169	7.07	2.16	0.09	0.20 **	0.15	0.06
Stimulating knowledge sharing	172	7.41	1.87	0.20 **	0.27 **	0.13	0.12
Giving colleagues feedback	171	7.32	1.93	0.27 **	0.27 **	0.15	0.14
Not wanting to do everything yourself	172	6.78	2.10	0.21 **	0.15 *	0.16 *	0.14
**External leader** (*α* = 0.68; ICC = 0.24)	-	6.69	1.78	0.30 **	0.38 **	0.01	0.19 *
Representing the team to the formal leader	165	7.17	1.82	0.25 **	0.34 **	0.11	0.07
Representing the team externally	152	7.08	2.25	0.24 **	0.23 **	0.07	0.23 *
Connecting with external teams	159	5.78	2.66	0.16 *	0.28 **	−0.08	0.08
**Logistics leader** (*α* = 0.66; ICC = 0.04)	-	6.71	1.78	0.21 **	0.19 *	−0.10	0.10
Paying attention to the logistics	146	7.06	2.27	0.07	0.14	−0.02	0.05
Internal housekeeping	123	5.84	2.74	0.20 *	0.16	−0.01	0.13
Paying attention to work safety	154	7.13	2.07	0.12	0.14	0.03	0.06
**Exemplary leader** (*α* = 0.70; ICC = 0.11)	-	7.06	1.59	0.32 **	0.26 **	0.20 *	0.18 *
Being vulnerable	173	6.72	2.23	0.30 **	0.24 **	0.19 *	0.13
Acting as a role model	171	7.43	1.73	0.19 *	0.17 *	0.17 *	0.09
Giving feedback in a calm, polite manner	171	6.98	2.06	0.25 **	0.20 *	0.09	0.18 *
**Unity leader** (*α* = 0.63; ICC = 0.04)	-	7.01	1.69	0.36 **	0.42 **	0.17 *	0.26 **
Emphasizing the common goal	166	6.97	2.05	0.32 **	0.42 **	0.20 *	0.24 **
Putting the team interests before own interests	168	7.01	1.93	0.27 **	0.25 **	0.08	0.12

Note. * *p* < 0.05; ** *p* < 0.01. *n* = number of respondents who rated the behavior and consequently deemed it relevant within their work context (instead of selecting the “not applicable” option). This number provides additional information regarding the prevalence of these behaviors in the workplace.

Overall, and in line with the conceptual model depicted in Figure 1, the multilevel regression modeling revealed that every leadership role contributed positively to the four outcomes. Each role predicted at least two outcomes, with job satisfaction ranking as the least predicted variable. Moreover, all functions contributed positively to at least one of the four outcomes, except for Coordinating tasks at team meetings (within *Task leadership*) and Daring to take initiative (within *Critical innovation leadership*). In some instances, all underlying functions of a given leadership role contributed to an outcome in a similar manner (e.g., the *Motivational leadership* functions for OCB). In contrast, certain roles exhibited specificity, wherein only specific functions appeared to be relevant for an outcome. Consequently, the combined effect of this role did not achieve significance. As an illustration, while the role of *External leadership* failed to attain significance for team cohesion (as shown in Table 4), this does not necessarily denote its ineffectiveness. Instead, this information implies that, for fostering team cohesion, the role of *External leadership* could potentially be redefined by focusing on the two pertinent underlying functions that demonstrated significant predictive power.

**Team effectiveness.** All 10 roles significantly contributed to team effectiveness. Given the potential variability in how teams conceptualize performance or team effectiveness based on the specific nature of their tasks, it may be worthwhile to investigate whether different leadership roles are important for different facets of team effectiveness. Thus, we conducted additional multilevel analyses, utilizing the seven distinct subscales of team effectiveness as outcome variables (i.e., output/quality/change/organization and planning/interpersonal/value/overall effectiveness). We did not find notable disparities in the impact of each leadership role across the different dimensions of team effectiveness. In fact, each role contributed to the prediction of each subscale, albeit with a few exceptions. Specifically, the *Connecting leader* and *Critical Innovation leader* roles did not significantly predict quality effectiveness, and the *External leader* role did not predict interpersonal effectiveness, possibly due to its emphasis on external networking rather than internal team dynamics. These findings are useful for organizations striving to achieve specific outcomes so that they can adapt their leadership roles accordingly. Overall, it is apparent that while the potency of effects might vary across roles, each role holds significance across almost every facet of team effectiveness. Moreover, the NCA unveiled a significant, medium effect for *Task leadership* (*p* = 0.045), indicating its status as a necessary condition for achieving a high level of team effectiveness. To elaborate, the bottlenecks depicted in Appendix E show that to attain 90% team effectiveness, an average score of 6.20 out of 10 is necessary for *Task leadership*. However, a score of 4.20 suffices for achieving 80%, suggesting that even a medium level of *Task leadership* holds substantial relevance for team effectiveness. Likewise, *Motivational leadership*, *Critical innovation leadership*, and *Exemplary leadership* emerged as necessary conditions for team effectiveness, each manifesting medium effects (all *p*’s < 0.05). These roles necessitate minimum scores of 7.00, 7.25, and 4.33, respectively, to achieve 90% of team effectiveness. Hence, the required level of *Motivational leadership* and *Critical innovation leadership* surpasses that required for *Exemplary leadership* or *Task leadership*.

At a deeper level, rooted in the regression analyses and NCA, four out of the five underlying functions of *Task leadership* predicted team effectiveness, with two functions (i.e., Representing the team internally and Creating clarity about each other’s work) also emerging as a necessary condition. Similarly, team effectiveness was predicted by four of the five functions of *Connecting leadership* and two out of three functions of *Social activity leadership* (i.e., Cultivating a good team atmosphere at work through social activities during work hours and Paying attention to special events of team members). Notably, all four functions belonging to *Motivational leadership* predicted all four outcomes, wherein Motivating colleagues to work and Recognizing the work of colleagues were found to be necessary conditions for team effectiveness. Among the functions of the *Critical innovation leader*, both Daring to take a critical look and initiating change and Preparing for the future were predictors and necessary conditions for achieving team effectiveness. It should be noted that the function Daring to take initiative within this role did not contribute to any of the four outcomes once the nested team structure was controlled for. Concerning *Team-development leadership*, three out of the four functions contributed to team effectiveness, with Giving colleagues feedback and Not wanting to do everything yourself being identified as necessary conditions. Likewise, team effectiveness was predicted by all three functions of *External leadership* and by two of the three functions of *Logistics leadership* (i.e., Internal housekeeping and Paying attention to work safety). In the case of *Exemplary leadership*, all three underlying functions contributed to team effectiveness with Being vulnerable surfacing as a necessary condition. Lastly, both underlying functions of *Unity leadership* predicted team effectiveness (with Putting the team interests before own interests as a necessary condition).

**OCB.** All 10 roles significantly predicted OCB. Remarkably, six roles (i.e., *Task leadership, Social activity leadership, Critical innovation leadership, Team-development leadership, External leadership*, and *Unity leadership*) were identified as necessary conditions (all medium effects and *p*’s < 0.05). To illustrate this, the bottleneck table in Appendix E indicates that to achieve 90% of OCB, *Unity leadership* must attain an average score of 7.00 out of 10. Moreover, *Motivational leadership* was a necessary condition for OCB, exhibiting a large effect size (*d* > 0.30 and *p* = 0.02). Here, an average score of 8.25 out of 10 was deemed requisite to achieve 90% of OCB.

At the level of functions, four of the five functions of *Task leadership* predicted OCB. Nearly all of these functions were identified as medium-sized necessary conditions for OCB, except for Coordinating tasks at team meetings. Similarly, concerning *Connecting leadership*, all five functions predicted OCB, and almost all functions (excluding Reducing tensions within the team) were necessary conditions for achieving OCB. Furthermore, OCB was predicted by all three functions of *Social activity leadership* (with Paying attention to special events of team members as a significant medium-sized necessary condition). All functions of *Motivational leadership* predicted OCB, with the function Recognizing the work of colleagues standing out as a necessary condition. Among the *Critical innovation leadership* functions, three out of four contributed to OCB, and all functions (except Daring to take initiative) were deemed necessary. All functions of *Team-development leadership* were predictive of OCB and also emerged as necessary conditions (except for Stimulating knowledge sharing). Likewise, all three functions of *External leadership* were linked to OCB, with Representing the team to the formal leader and Connecting with external teams being identified as necessary conditions. For *Logistics leadership*, two out of three functions showed a predictive relation, and all three functions of *Exemplary leadership* (with Being vulnerable as a necessary condition) and both functions of *Unity leadership* predicted OCB.

**Job satisfaction.** Five leadership roles significantly predicted job satisfaction (i.e., *Task leader*, *Social activity leader*, *Team-development leader*, *Unity leader*, and *Exemplary leader*). However, none of these roles qualified as a necessary condition for job satisfaction (all *d*’s < 0.10 and *p* > 0.05). Only two of five functions of *Task leadership* contributed to job satisfaction (i.e., Creating clarity about each other’s work and Monitoring the work (the latter also being identified as a necessary condition)). Interestingly, none of the functions underlying *Connecting leadership* and only one of the three functions of *Social activity leadership* (i.e., Cultivating a good team atmosphere at work through social activities during work hours) was found to be a predictor of job satisfaction. In contrast, all four functions of *Motivational leadership* were significant predictors, along with two of the four functions of *Critical innovation leadership* (i.e., Daring to take a critical look and initiating change, and Preparing for the future). All functions within *Team-development leadership* predicted job satisfaction (with Not wanting to do everything yourself as a necessary condition), together with two of the three functions of *Exemplary leadership* (i.e., Acting as a role model and Being vulnerable (which was also a necessary condition)). Here, too, both functions of *Unity leadership* contributed to job satisfaction, while none of the functions within *External leadership* and *Logistics leadership* demonstrated such a predictive relation.

**Team cohesion.** Team cohesion was significantly predicted by the same five roles that predicted job satisfaction, in addition to the *Connecting leader*, *Motivational leader*, and *Critical innovation leader*. Nonetheless, as with job satisfaction, none of these roles emerged as a necessary condition for team cohesion (all *d*’s < 0.10 and *p*’s > 0.05). At the function level, only two of five *Task leadership* functions (i.e., Ensuring a fair division of tasks and Creating clarity about each other’s work) and one of five *Connecting leadership* functions (i.e., Ensuring connection and interaction within the team) predicted team cohesion. *Social activity leadership* had two relevant functions (i.e., Organizing social activities outside work hours and Paying attention to special events of team members), alongside all four functions of *Motivational leadership*. Conversely, none of the functions within *Critical innovation leadership* and *Logistics leadership* proved to be significant predictors for team cohesion, unlike the leadership roles themselves. However, two of the four *Team-development leadership* functions (i.e., Giving colleagues feedback and Not wanting to do everything yourself), as well as two of the three *External leadership* functions (i.e., Connecting with external teams and Representing the team externally (also a necessary condition)) predicted team cohesion. Finally, only one out of three *Exemplary leadership* functions (i.e., Giving feedback in a calm, polite manner) and one out of two *Unity leadership* functions (i.e., Emphasizing the common goal) had significant predictive power for team cohesion.

### 3.2. Aim 2: The Moderating Role of Team-Specific Characteristics

Recognizing that the efficacy of shared leadership may not be uniform across various team contexts, we introduced three moderator variables to explore potential variations in these contexts. Specifically, we examined the moderating impact of team size (H1), team tenure (H2), and team identification (H3) on the relation between each peer leadership role and the four work outcomes (see Figure 2). As depicted in Table 6, the results confirmed H1, H2, and H3 to a certain extent, as moderating effects were identified for specific leadership roles (i.e., *Unity leadership* for H1 and H3; *External leadership* for H2) but not the entire set of roles. As a result, many of the significant relations unveiled in Aim 1 seemed to hold consistent across teams, irrespective of their size, tenure, or shared team identification. Nevertheless, it is important to exercise caution while interpreting the moderating role of team size and team tenure, given that the interaction term’s regression coefficients were relatively low, suggesting a relatively small effect size.

**Figure 3:**
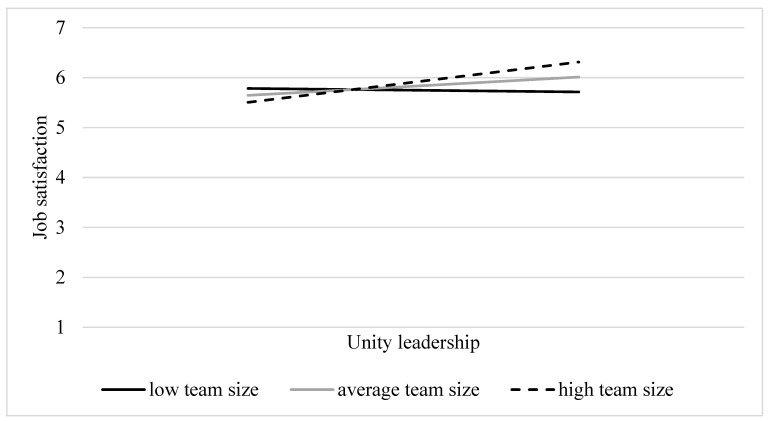
Interaction of *Unity leadership* and team size with regard to job satisfaction.

**Team size.** The relation between *Unity leadership* and job satisfaction was moderated by team size (*β* = 0.27, *SE* = 0.09, *p* = 0.003). However, in contrast to H1, the results from simple slope analyses (see Figure 3) revealed that *Unity leadership* predicted job satisfaction more strongly when the team comprised a larger number of members (*β* = 0.24, *SE* = 0.06, *p* < 0.001) as compared to an average number of members (*β* = 0.11, *SE* = 0.04, *p* < 0.001). Notably, in teams with a smaller membership size, this relation became nonsignificant (*β* = −0.02, *SE* = 0.06, *p* = 0.73). Hence, in teams with fewer members, the appointment of this particular leadership role to a peer leader appears to be less important. This finding contradicts our initial hypothesis, which posited that the influence of peer leadership on outcomes would be stronger in teams with fewer members.

**Figure 4:**
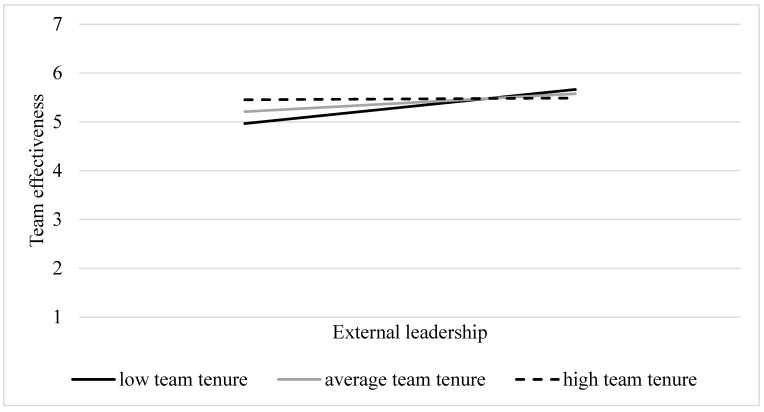
Interaction of *External leadership* and team tenure with regard to team effectiveness.

**Team tenure.** Team tenure moderated the relation between *External leadership* and team effectiveness (*β* = −0.24, *SE* = 0.08, *p* = 0.006), although this interaction effect might not be particularly strong given the relatively low value of the regression coefficient. The results of simple slope analyses (see Figure 4) indicated that in accordance with H2, *External leadership* predicted team effectiveness more strongly when the level of team tenure was at the lower end (*β* = 0.20, *SE* = 0.04, *p* < 0.001) compared to an average level of team tenure (*β* = 0.10, *SE* = 0.03, *p* < 0.001). Conversely, for teams with higher levels of team tenure, this relation did not prove significant (*β* = 0.01, *SE* = 0.05, *p* = 0.83). Notably, no significant interaction effects were identified in relation to OCB, job satisfaction, and team cohesion. These results align with our hypothesis that the relations would be more pronounced in teams with shorter rather than longer tenures.

**Figure 5:**
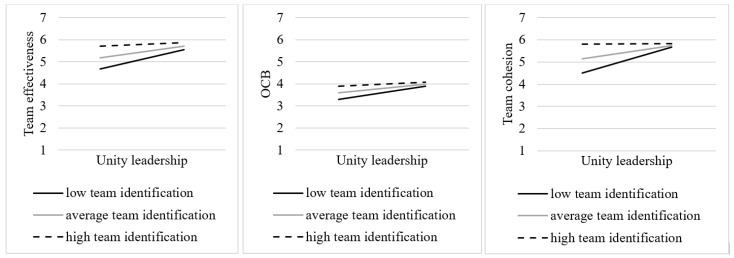
Interaction of *Unity leadership* and team identification on team effectiveness (**left**), OCB (**middle**), and team cohesion (**right**).

**Team identification**. Team identification (*M* = 5.64, *SD* = 0.65) significantly moderated the relation between *Unity leadership* and three outcomes: team effectiveness (*β* = −0.14, *SE* = 0.06, *p* = 0.005), OCB (*β* = −0.16, *SE* = 0.06, *p* = 0.011), and team cohesion (*β* = −0.26, *SE* = 0.06, *p* = 0.001). Subsequent simple slope analyses (see Figure 5) unveiled that in contrast to H3, *Unity leadership* predicted team effectiveness more strongly in teams with a lower degree of team identification (*β* = 0.25, *SE* = 0.05, *p* < 0.001) compared to teams with an average level of team identification (*β* = 0.15, *SE* = 0.03, *p* < 0.001). This relation turned nonsignificant in teams with higher levels of team identification (*β* = 0.05, *SE* = 0.04, *p* = 0.25). Likewise, *Unity leadership* predicted OCB more strongly in teams with lower levels of team identification (*β* = 0.18, *SE* = 0.04, *p* < 0.001) as opposed to teams with an average degree of team identification (*β* = 0.12, *SE* = 0.02, *p* < 0.001). This relation ceased to be significant in teams with higher levels of team identification (*β* = 0.05, *SE* = 0.03, *p* = 0.051). Finally, simple slope analyses revealed that *Unity leadership* exerted a stronger predictive influence on team cohesion in teams with lower levels of team identification (*β* = 0.34, *SE* = 0.08, *p* < 0.001) as compared to teams with an average level of team identification (*β* = 0.17, *SE* = 0.04, *p* < 0.001). This relation lost significance in teams with higher levels of team identification (*β* = 0.05, *SE* = 0.06, *p* = 0.93; see Figure 5). Contrary to our expectations, the *Unity leader* appears to be only effective in bolstering team effectiveness, OCB, and team cohesion when team identification is relatively low.

While we treated team identification as a team-level construct, we acknowledge the potential variability in the perceived levels of team identification among individual team members. Therefore, we explored whether the moderation effects based on individual team members’ scores on team identification diverged from those based on aggregate scores. Using individual scores, we identified identical interaction effects between *Unity leadership* and the outcomes of team effectiveness, OCB, and team cohesion. To elaborate, our simple slope analyses showed that when team identification levels were low, the impact of *Unity leadership* on team effectiveness became more pronounced (*β* = 0.18, *SE* = 0.04, *p* < 0.001) in comparison to teams with an average level of team identification (*β* = 0.11, *SE* = 0.03, *p* < 0.001). This relation lost its statistical significance for teams with higher levels of team identification (*β* = 0.04, *SE* = 0.04, *p* = 0.29). Similarly, *Unity leadership* predicted both OCB and team cohesion more strongly in teams with low levels of team identification (*β* = 0.14, *SE* = 0.03, *p* < 0.001 and *β* = 0.28, *SE* = 0.06, *p* < 0.001, respectively) than in teams with an average level of team identification (*β* = 0.09, *SE* = 0.02, *p* < 0.001 and *β* = 0.11, *SE* = 0.04, *p* = 0.004). Again, these relations became nonsignificant when team identification levels were high (*β* = 0.04, *SE* = 0.03, *p* = 0.11 and *β* = −0.05, *SE* = 0.05, *p* = 0.36).

Nonetheless, the utilization of individual team identification scores uncovered two additional interactions. First, we found a significant interaction effect of team identification in the relation between *Social activity leadership* and team effectiveness (*β* = −0.07, *SE* = 0.03, *p* = 0.007). The simple slope analysis indicated that when an individual’s team identification was low, *Social activity leadership* predicted team effectiveness more strongly (*β* = 0.14, *SE* = 0.04, *p* < 0.001) as compared to cases with an average level of team identification (*β* = 0.07, *SE* = 0.03, *p* = 0.012). For individuals with higher levels of team identification, this relation became nonsignificant (*β* = −0.01, *SE* = 0.04, *p* = 0.86). Second, the moderation effect of an individual’s team identification was also evident in the relation between *Exemplary leadership* and team effectiveness (*β* = −0.05, *SE* = 0.03, *p* = 0.045). Interestingly, *Exemplary leadership* predicted team effectiveness regardless of the level of team identification. However, this effect was stronger when individuals reported an average level of team identification (*β* = 0.15, *SE* = 0.03, *p* < 0.001) or, albeit less strong, a lower level of team identification (*β* = 0.21, *SE* = 0.04, *p* < 0.001) compared with individuals perceiving higher levels of team identification (*β* = 0.09, *SE* = 0.04, *p* = 0.025). Here, too, caution is needed in interpreting these findings due to the relatively small effect sizes.

## 4. Discussion

Establishing a sustainable well-being–productivity synergy requires leadership behaviors that emphasize both the well-being and performance of employees. In this study, our first aim was to examine how distinct peer leadership roles, encompassing their inherent leadership functions, enhance both aspects and contribute to this synergy in terms of four outcomes: team effectiveness, OCB, job satisfaction, and team cohesion. Our results showed that each of the 10 leadership roles contributed positively to at least two of the four outcomes, underscoring their significance within organizational contexts. Thus, it appears that leaders within a team demonstrate the capability to take on diverse peer leadership roles, with these roles varying in their effectiveness in enhancing well-being and performance outcomes, ultimately contributing to the cultivation of a sustainable work environment. This finding holds four conclusions for the advancement of shared leadership theory (and peer leadership in particular). First, it adds to the growing number of empirical studies that demonstrate the impactful role of peer leaders in shaping work outcomes (e.g., [74]). This aligns with the functional leadership framework that posits that beyond the formal leader, team members have the potential to embrace leadership roles and thereby cultivate favorable outcomes [36,75]. Second, our results provide preliminary quantitative support for the 10 peer leadership roles identified by Edelmann et al. [12]. They offer a nuanced picture of the roles and functions that are most relevant to be fulfilled by peer leaders to yield specific outcomes, thereby optimizing shared leadership implementations in practice.

Third, in search of a more fine-grained analysis, our study revealed that not all leadership roles and their corresponding functions held equal significance across various outcomes within our sample. This observation resonates with the theoretical framework of role composition, which delves into the differential impact of distinct roles within a team [76]. Some roles had a stronger or weaker effect on an outcome relative to others. To illustrate, the roles of *Task leader* and *Motivational leader* showed a stronger relation with job satisfaction in comparison to the roles of *Connecting leader* and *External leader*. This finding supports our notion that the efficacy of shared leadership structures may depend on the specific leadership content being shared, thereby offering insight into the inconsistent findings pervading the shared leadership literature. Fourth, our study heeded scholars’ call to extend investigations beyond performance outcomes alone [38,77]. Hereby, we demonstrated that distinct peer leadership roles not only predicted team effectiveness and OCB but also job satisfaction and team cohesion.

While achieving these outcomes is a goal in itself, each examined variable is likely to contribute to employee well-being and hence a sustainable workforce. To begin, effective teams are characterized by streamlined workflows, improved communication, and optimized task distribution. This collective efficacy can alleviate the workload burden on individual employees and provide them with a sense of achievement and social support that helps buffer work stress [78,79]. Similarly, when employees engage in OCB, it can cultivate a positive team climate and a sense of purpose, both of which contribute to employee well-being [42]. Next, heightened job satisfaction makes employees more engaged and committed, thereby positively affecting their overall well-being (e.g., reduced burnout) [80]. Lastly, a strong team cohesion nurtures a sense of belonging, collaboration, and support, resulting in a positive work environment and reduced stress [39].

When interpreting our results with respect to Aim 1, we stress the importance of exercising caution. Specifically, we aggregated leadership behaviors (i.e., functions) that shared conceptual similarity into a comprehensive meta-construct (i.e., a leadership role). The flaw of synthesizing a role as an average of its constituent functions is that it presupposes uniform fulfillment of each function. However, such a uniform fulfillment might not be the actual scenario; some excellent leaders might adeptly fulfill a majority of these underpinning functions while potentially falling short in others. Moreover, our results demonstrate differentiating findings for specific functions within roles, wherein some functions predicted an outcome, while others did not. These insights could be used to redefine the content of a role when targeting a specific outcome.

Nevertheless, the practice of consolidating functions into overarching leadership roles when implementing a leadership structure can still be a powerful method. This can be attributed to the cognitive phenomenon wherein individuals tend to remember ideas better when presented under a higher-order label [81]. Thus, bundling diverse functions together “underneath a single umbrella term highlighting their commonalities can make training and development more efficient and effective” [82] (p. 83). Hence, it remains important to bear in mind that not all functions might be equally relevant for achieving a desired effect, and thus the definitions of these roles may need to be revisited.

Our second aim was to investigate the potential moderating influences inherent in three team-specific characteristics: team size, team tenure, and team identification. Our findings underscore that the majority of relations identified in Aim 1 remained consistent across teams, irrespective of their size (H1), tenure (H2), or the extent of shared team identification (H3). This finding suggests that, overall, the magnitude of potential effects of the 10 peer leadership roles on the four outcomes is not contingent on specific team characteristics. However, for specific relations, these team characteristics did seem to influence the efficacy of peer leadership roles.

First, we found that *Unity leadership* exclusively predicted job satisfaction within larger teams. This finding could be attributed to the notion that as team size increases, team members may experience more interpersonal distance and greater ambiguity in team objectives [8,47]. Consequently, team coordination could suffer, leading to a sense of discontent. In larger teams, the presence of an individual who emphasizes the collective team goal and places their own interests secondary to this could be especially important for maintaining job satisfaction among team members.

Second, teams characterized by longer durations of collaboration did not seem to profit from the *External leadership* role in achieving heightened levels of team effectiveness. This finding aligns with our initial expectations and can be rationalized by considering that newly established teams with peer leaders encounter a reduced risk of power struggles or tensions that could harm team performance [50]. Also, teams with lower tenure may have a greater need for an individual who represents their team and fosters connections with other teams, given that individual team members may not yet have had the opportunity to establish such connections. In contrast, teams with longer tenures may have already formed external links over time and thus may not require external representation to the same extent as less mature teams.

Third, we observed that the role of the *Unity leader* was relevant for team effectiveness, OCB, and team cohesion only within teams characterized by a relatively lower average level of team identification. This finding concurs with the substitutes for leadership theory [83], indicating that *Unity leadership* held significance only in contexts where team identification was lacking. Evidently, it is precisely those teams whose members do not strongly identify with their collective unit that profit from adopting a shared leadership structure, resulting in potential advancements. The presence of an individual within their team who highlights commonalities and acts in the group’s interests can catalyze team members’ recognition of shared values (ultimately bolstering team cohesion) and motivate them to actively contribute (thus enhancing OCB and team effectiveness). In contrast, teams characterized by a strong sense of team identification might already possess an intrinsic motivation to align with common goals [84]. Although the appointment of a dedicated *Unity leader* in such teams might not pose any harm, its impact on achieving outcomes could be comparatively negligible.

Fourth, based on individual perceptions of team identification, we also found that the *Social activity leader* role predicted team effectiveness solely within teams whose members indicated a relatively low level of team identification. A plausible interpretation of this finding is that for an individual who does not strongly identify with the team, organizing social activities within the team is particularly crucial in fostering a sense of connection with the team and acquainting oneself with colleagues, thereby enhancing coordination processes and improving team effectiveness.

Finally, the role of *Exemplary leadership* appeared as most influential in predicting team effectiveness when individual team members reported either a relatively low or an average level of team identification. In contrast, the impact was comparatively weaker for those team members who strongly identified with their team. The exemplary behavior of a fellow team member (e.g., demonstrating a positive work ethos and feedback in a polite manner) might hold even greater significance for a team member who lacks a strong sense of identification with their team. This is because such behavior elucidates the prevailing norms and values embraced by the team, and this heightened clarity can bolster team effectiveness.

In light of these findings, our study contributes to the understanding that the relation between shared leadership and outcomes may, in some instances, be moderated by other variables (e.g., [9,85]). Apparently, shared leadership cannot be uniformly applied, as its benefits may not always be found across different types of teams.

### 4.1. Strengths, Limitations, and Suggestions for Future Research

One notable strength of this study lies in the approach we employed when soliciting participant responses. Rather than inquiring about the perceived importance of specific leadership functions, we directly inquired about the extent to which these functions were present in their work environments. This methodological choice allowed us to establish a more objective link between leadership functions and their subsequent relationship with outcomes. As a result, our conclusions regarding the significance of each function are grounded in a more neutral and empirical assessment, thus circumventing the subjective bias associated with perceived importance.

Furthermore, this is the first study to apply Necessary Condition Analysis as a novel research method to empirically evaluate the influence of distinct peer leadership roles on work-related outcomes. This analytical approach offers a unique advantage by providing insights that complement those derived from conventional methods such as regression analysis, thereby paving the way for further research in the realm of organizational behavior and, specifically, leadership. In terms of ecological validity, our study benefits from a sample that represents a diverse cross-section of the work population. This diversity is attributed to our meticulous participant recruitment process, which included individuals from various sectors and industries, and a rigorous stratification scheme that accounted for hierarchy status and the type of organization.

However, this study is not without its limitations. From a methodological standpoint, the primary limitation of our study is the reliance on a cross-sectional research design. Consequently, we cannot rule out the possibility that the observed relations could be reversed (e.g., team effectiveness acting as not only an outcome but also a precursor to peer leadership). Prior research has shown that variables conventionally regarded as outcome variables were found to influence hierarchical differentiation within a team. For example, antecedents such as past performance and social support among team members have been demonstrated to increase and decrease the extent of hierarchical differentiation [86,87]. To ascertain whether shared leadership yields different outcomes, we encourage researchers to conduct intervention studies wherein peer leaders can undergo training in a specific leadership role. Although no causality can be inferred from our data, the results nonetheless provide initial evidence for the significance of distinct peer leadership roles and their respective functions in promoting a supportive and sustainable work environment.

Second, due to the multilevel structure of our dataset, we acknowledge the inherent limitations in the statistical power of our moderation tests. The relatively low number of teams, in combination with the extensive number of separate analyses conducted (i.e., 10 predictors and four outcome variables) probably led to less robust results (e.g., inaccurate estimations of standard errors) [88]. To consolidate our findings, future research should include a larger pool of teams to enhance statistical power and improved generalizability to broader populations, especially in the context of multi-level models. Additionally, our findings exclusively offer insights into the individual relevance of each leadership role, as assessed through simple regression analysis. While this initial study aimed to establish a foundation in peer leadership research, the next step should involve the exploration of the interplay among these roles and their combined influence. There is a possibility that some of the leadership roles outlined by Edelmann et al. [12] have interdependent relationships. For instance, Mumford et al. [89] propose that technical expertise and creative thinking skills work in tandem to drive innovation. Similarly, one might conjecture that the roles of *Logistics leader* and *Critical Innovation leader* jointly contribute to problem-solving processes. Qualitative Comparative Analysis (QCA) [90] presents a viable avenue for delving into the configurations of leadership roles that foster the desired outcomes, thereby identifying the most relevant set of leadership roles to be fulfilled in conjunction with one another. One of the many advantages of this method is the ability to ascertain multiple causal pathways and configurations for both high and low levels of the same outcome (i.e., equifinality and causal asymmetry) [91].

A third limitation of our study lies in the method through which we gauged peer leadership. Specifically, we asked participants about the behaviors exhibited by peer leaders within their respective teams (comprising one or more team members). For that reason, our study lacks precision in terms of discerning the exact number of peer leaders within a team and the specific roles they undertook. To address this gap, future research should identify the most efficient structure (e.g., the optimal number of peer leaders per role) for organizational teams. In this context, we advocate for the adoption of Social Network Analysis (SNA) as a valuable tool to unravel the leadership structure within a team (e.g., network density) [8,92,93]. This technique allows researchers to explore whether the 10 roles can best be fulfilled by a solitary peer leader, a select ensemble of peer leaders, or all team members collectively. In addition, by analyzing the intercorrelations among distinct role networks, SNA reveals patterns indicating whether specific content-related roles consistently coincide with the same individuals. If so, it becomes plausible to treat these roles as a singular entity in future studies. These analyses can yield a more nuanced contribution to the theoretical conceptualization of shared leadership as well as enhance its practical implementation.

On a related note, it is important to acknowledge the inherent dynamic nature of leadership structures, which can evolve as teams and their situational needs change rapidly [94]. While our study offered a cross-sectional snapshot of the prevailing leadership structure within teams, the following step in research entails delving into the dynamic progression of shared leadership across time, for example, using longitudinal study designs with the regular application of network analytic techniques [95].

As a fourth limitation, scholars agree that both vertical and shared leadership are important in bolstering team functioning [96]. Hence, the role of formal leaders also warrants consideration. It is essential to recognize that team members assuming leadership roles does not render formal leadership authority obsolete. On the contrary, formal leaders can play a crucial role in instigating, facilitating, and orchestrating shared leadership structures (e.g., helping peer leaders develop their leadership potential). Moreover, teams may need to shift between these two leadership sources in diverse situations or phases, with vertical leadership proving more advantageous in times of change, crisis, or pressure [7]. To avoid the risk of making this study overly complex, we intentionally focused exclusively on peer leaders as the source of leadership. This deliberate focus allowed us to delve deeper into the nuances of peer leadership. Nevertheless, we acknowledge that both sources of leadership can coexist and operate in tandem [7]. To gain a more comprehensive picture of leadership within teams, future research should explore the interaction between peer leadership functions and formal leadership functions. Thereby, insights can be yielded into whether certain roles are optimally fulfilled by either or both sources of leadership, shedding light on whether it suffices for a role to be assumed by a single source among the two. As demonstrated by the points above, before the practical implementation of these roles (such as within leadership development programs), more research is needed to validate their distinctiveness and independence concerning the different outcomes.

### 4.2. Theoretical Contributions and Practical Implications

Taken together, the insights gained from our study contribute to three distinct literature streams. First, they enrich organizational behavior research by providing a clearer understanding of how specific employee behaviors contribute to the promotion of employee well-being at work, alongside other favorable outcomes for the team and organization. Second, our research extends the field of sustainability management by illustrating which precise behaviors of peer leaders should be cultivated and further developed to achieve social sustainability in the workplace. Third, our findings advance the existing (shared) leadership literature. By adopting a function-based approach, we enhance our understanding of how shared leadership structures can be implemented by identifying key leadership behaviors that peer leaders can fulfill to attain diverse work outcomes. Specifically, we investigated the actions peer leaders can take to enhance outcomes that extend beyond mere team performance and how these actions may vary across different teams.

Incorporating the roles and functions explored in this study into daily business operations can enhance organizational practices. While the set of peer leadership roles remains potentially subject to revision (e.g., some roles may need to be combined), the findings underscore the significance of the diverse peer leadership roles defined by Edelmann et al. [12] in generating performance- and well-being/relationship-oriented outcomes. As such, we have demonstrated that organizational behavior in the form of specific peer leadership roles and functions link with both well-being and performance outcomes, which can promote a ‘sustainable well-being–productivity synergy’. This knowledge bears practical relevance, particularly in light of the prominent role that (mental) health has assumed within the framework of the 17 United Nations Sustainable Development Goals defined by UN member states in 2015 [97]. One of these goals directly relates to employee well-being at work (i.e., SDG-3), delineating explicit objectives for organizations to foster and sustain a secure and supportive work environment, encompassing employees’ physical and mental well-being.

Recognizing the (direct or indirect) influence of various peer leader behaviors on employee well-being can offer additional foundations for tailored shared leadership programs, contingent on the organization’s and its employees’ specific needs, as well as on the characteristics of the team setting, such as its size. More specifically, managers or practitioners can integrate these roles and functions into their strategies, selection processes, and performance metrics, thereby enhancing leadership training and development in the context of shared leadership. The influence of peer leadership may be more readily realized when the functions are observable and meaningful (i.e., are important and/or necessary for obtaining an outcome) [23]. Hence, this set of peer leadership roles can establish a framework that can facilitate team members’ navigation through the diverse behaviors when officially assigning responsibilities to peer leaders.

Human resource management departments can organize training workshops to assist peer leaders in comprehending specific behavioral changes that could enhance their effectiveness in achieving desired outcomes. These workshops could go beyond the existing ones, which concentrate on more broadly defined leadership concepts (e.g., leadership styles). A facilitator can initially elucidate the distinct roles, their underlying functions, and their relevance to desired outcomes within the specific context. In the next step, managers or team members can select the roles they perceive as most relevant in their context. Armed with this knowledge, the team can be encouraged to focus on the roles that are relevant to achieving this specific outcome. If the priority is establishing team cohesion within a newly established team, appointing peer leaders to fulfill the roles of *Social activity leadership* and *Unity leadership* may be especially impactful. Next, the workshop can further delve into the function (i.e., behavioral) level to decide how these roles will manifest concretely. For instance, a particular team may find that only two out of the five *Task leadership* functions are of significant relevance. These specific functions may then receive attention throughout implementation. In this way, managers can flexibly deploy distinct leadership roles into their practices. To identify the most qualified team members for specific roles, the *Shared Leadership Mapping* by Fransen et al. [93] may be helpful. This technique uses social network analysis based on team members’ perceptions to map the leadership structure of the team. By strategically allocating key roles among designated peer leaders, teams can ultimately enhance employee well-being and cultivate a sustained human capital advantage.

## 5. Conclusions

While prior research has highlighted the positive impact of peer leaders on outcomes, it remains unclear which specific peer leadership behaviors are able to cultivate a supportive and sustainable work environment. In the pursuit of facilitating the implementation of shared leadership, our first aim was to scrutinize the relations between the leadership roles and functions, as outlined by Edelmann et al. [12], and four key outcomes: job satisfaction, team cohesion, team effectiveness, and OCB. The variability in these relations suggests that certain roles may be more important for specific outcomes than others. Additionally, we found that only specific relations were moderated by team size, team tenure, and team identification. These results offer a more nuanced understanding of shared leadership, shedding light on which roles and functions bear particular relevance and/or necessity in achieving targeted outcomes. To this end, this research further contributes to the theoretical discussions on organizational behavior and well-being at work, while also providing practical insights for achieving sustainable well-being and productivity in the workplace in the context of shared leadership.

## Figures and Tables

**Table 1:** Overview of the 10 peer leadership roles and their respective definitions as identified by Edelmann et al. [12].

Peer Leadership Role (Identified by Edelmann et al. [12])	Definition of Peer Leadership Role (Provided by Edelmann et al. [12])
Task Leader	The Task leader ensures a fair allocation of tasks among team members, tracking the progress of the work schedule, and taking the lead during team gatherings. Additionally, this leader provides colleagues with a deeper understanding of one another’s expertise and tasks, while clarifying the interconnections among their respective tasks.
Connecting Leader	The Connecting leader is aware of the colleagues’ well-being and is regarded as a confidant. This leader facilitates connections among different team members, builds linkages between various subgroups, and takes the lead in resolving tensions that may arise between colleagues.
Social activity Leader	The Social activity leader ensures a harmonious atmosphere within the team. This leader remains attentive to significant events (such as colleagues’ birthdays) and assumes initiative in organizing both intra- and extramural social activities for the team.
Motivational Leader	The Motivational leader motivates colleagues, acknowledges their contributions, and expresses appreciation. In addition, this leader encourages other team members to proactively take the lead and articulate their opinion.
Critical Innovation Leader	The Critical innovation leader adopts a critical view of the team aimed at instigating change. This leader takes the lead in instigating initiative and prepares the team to navigate future endeavors adeptly.
Team-development Leader	The Team-development leader facilitates the continued growth of colleagues in their respective areas of expertise. Furthermore, this leader fosters a culture of knowledge sharing within the team, dares to provide constructive feedback to colleagues, and provides guidance to new team members as they embark on their professional journey.
External Leader	The External leader represents the team both to the top of the organization and to external parties (e.g., clients, media). Moreover, this leader encourages connectivity and collaborative efforts between their own team and other teams within the organization.
Logistics Leader	The Logistics leader ensures the availability of essential equipment and maintains a clean working environment. This leader is also responsible for upholding compliance with safety guidelines.
Exemplary Leader	The Exemplary leader exemplifies leadership by demonstrating a positive work ethic, delivering feedback to colleagues in a composed and courteous manner, and maintaining optimism during both changes and setbacks. Additionally, this leader is willing to display vulnerability, for example, by seeking assistance from colleagues when necessary.
Unity Leader	The Unity leader actively seeks a common goal, motivates colleagues within the team to prioritize this goal, and consistently places the team’s interests ahead of their personal interests.

**Table 2:** Demographic information of participants and their team.

	Formal Leaders (*n* = 31)		Team Members (*n* = 152)	
**Gender**				
Male	20		77	
Female	11		72	
**Education level**				
Low (professional education at most)	7		49	
High (university degree at least)	24		102	
	***M***	***SD***	***M***	***SD***
Age (in years)	45.29	9.57	41.42	11.25
Years working in current function	10.51	10.96	7.34	2.38
Organizational tenure (in years)	15.30	12.03	10.66	9.97
General leadership experience (in years)	12.79	9.22	n.a.	
Collaboration with formal leader (in years)	n.a.		4.27	5.31
Team tenure (in years)	6.98	8.09	5.53	5.61

Note. n.a. = not applicable.

**Table 4:** Fixed effects of the multilevel regression modeling for all four outcomes with leadership role/function as defined by Edelmann et al. [12] as level 1 predictor and a random intercept to control for the nested nature of the data.

Leadership Roles and Their Underlying Functions (Defined by Edelmann et al. [12])	Team Effectiveness *β* (*SE*)	OCB *β* (*SE*)	Job Satisfaction *β* (*SE*)	Team Cohesion *β* (*SE*)
**Task leader**	0.39 *** (0.08)	0.39 *** (0.07)	0.36 * (0.08)	0.20 * (0.09)
Ensuring a fair division of tasks	0.31 *** (0.07)	0.30 *** (0.07)	0.14 (0.08)	0.18 * (0.08)
Monitoring the work	0.27 *** (0.07)	0.28 *** (0.07)	0.28 *** (0.08)	0.10 (0.08)
Representing the team internally	0.26 *** (0.07)	0.31 *** (0.11)	0.09 (0.08)	0.12 (0.08)
Creating clarity about each other’s work	0.38 *** (0.07)	0.38 *** (0.07)	0.24 ** (0.08)	0.25 ** (0.08)
Coordinating tasks at team meetings	0.12 (0.08)	0.13 (0.08)	−0.02 (0.10)	0.09 (0.08)
**Connecting leader**	0.23 ** (0.07)	0.31 *** (0.07)	0.15 (0.08)	0.22 * (0.09)
Being a confidential advisor for colleagues	0.18 * (0.07)	0.24 *** (0.07)	0.13 (0.07)	0.09 (0.08)
Cultivating a healthy work culture	0.24 *** (0.07)	0.25 *** (0.07)	0.08 (0.08)	0.04 (0.04)
Reducing tensions within the team	0.17 * (0.07)	0.23 ** (0.07)	0.14 (0.08)	0.15 (0.08)
Opening up to colleagues	0.12 (0.07)	0.18 * (0.07)	0.10 (0.08)	0.15 (0.08)
Ensuring connection and integration within the team	0.20 ** (0.07)	0.28 *** (0.07)	0.14 (0.08)	0.18 * (0.08)
**Social activity leader**	0.26 *** (0.08)	0.39 *** (0.07)	0.11 (0.08)	0.23 ** (0.09)
Cultivating a good team atmosphere at work through social activities during work hours	0.21 ** (0.07)	0.26 *** (0.11)	0.19 * (0.07)	0.15 (0.08)
Organizing social activities outside work hours	0.14 (0.08)	0.33 *** (0.08)	0.04 (0.09)	0.21 * (0.09)
Paying attention to special events of team members	0.21 ** (0.07)	0.29 *** (0.07)	0.03 (0.08)	0.16 * (0.08)
**Motivational leader**	0.37 *** (0.07)	0.29 *** (0.07)	0.23 ** (0.07)	0.21 ** (0.08)
Motivating colleagues to work	0.29 *** (0.07)	0.23 *** (0.07)	0.17 * (0.07)	0.16 * (0.08)
Encouraging colleagues to take initiative and voice their opinion	0.31 *** (0.06)	0.22 *** (0.07)	0.20 ** (0.07)	0.16 * (0.08)
Recognizing the work of colleagues	0.31 *** (0.06)	0.24 *** (0.07)	0.21 ** (0.07)	0.23 ** (0.08)
Expressing gratitude	0.28 *** (0.07)	0.25 *** (0.07)	0.19 ** (0.07)	0.17 * (0.08)
**Critical innovation leader**	0.23 ** (0.07)	0.31 *** (0.07)	0.15 (0.08)	0.22 * (0.09)
Daring to take a critical look and initiating change	0.32 *** (0.07)	0.34 *** (0.07)	0.19 * (0.08)	0.13 (0.08)
Daring to take initiative	0.13 (0.07)	0.13 (0.07)	0.08 (0.08)	0.08 (0.08)
Daring to criticize the formal leader	0.06 (0.08)	0.19 ** (0.07)	−0.03 (0.08)	0.04 (0.08)
Preparing for the future	0.27 *** (0.07)	0.30 *** (0.07)	0.22 ** (0.08)	0.05 (0.08)
**Team-development leader**	0.28 *** (0.07)	0.33 *** (0.07)	0.23 ** (0.07)	0.19 * (0.08)
Developing the growth and expertise of colleagues	0.12 (0.07)	0.25 *** (0.07)	0.17 * (0.07)	0.07 (0.08)
Stimulating knowledge sharing	0.22 ** (0.07)	0.21 ** (0.07)	0.16 * (0.07)	0.12 (0.08)
Giving colleagues feedback	0.30 *** (0.07)	0.31 *** (0.07)	0.19 * (0.07)	0.17 * (0.08)
Not wanting to do everything yourself	0.24 *** (0.07)	0.19 ** (0.07)	0.16 * (0.08)	0.19 * (0.08)
**External leader**	0.30 *** (0.08)	0.37 *** (0.08)	0.06 (0.09)	0.14 (0.09)
Representing the team to the formal leader	0.24 ** (0.07)	0.33 *** (0.07)	0.13 (0.08)	0.05 (0.08)
Representing the team externally	0.24 ** (0.07)	0.20 ** (0.08)	0.14 (0.08)	0.22 ** (0.08)
Connecting with external teams	0.16 * (0.08)	0.25 *** (0.07)	−0.05 (0.08)	0.04 * (0.09)
**Logistics leader**	0.27 *** (0.08)	0.21 ** (0.08)	0.06 (0.09)	0.11 (0.09)
Paying attention to the logistics	0.13 (0.07)	0.19 ** (0.07)	0.02 (0.08)	0.08 (0.08)
Internal housekeeping	0.24 ** (0.08)	0.15 (0.08)	0.06 (0.09)	0.14 (0.09)
Paying attention to work safety	0.17 * (0.07)	0.19 ** (0.07)	0.06 (0.08)	0.06 (0.08)
**Exemplary leader**	0.36 *** (0.07)	0.28 *** (0.07)	0.24 ** (0.07)	0.21 ** (0.08)
Being vulnerable	0.31 *** (0.07)	0.22 ** (0.07)	0.23 ** (0.07)	0.14 (0.08)
Acting as a role model	0.23 *** (0.07)	0.18 * (0.07)	0.21 ** (0.07)	0.10 (0.08)
Giving feedback in a calm, polite manner	0.29 *** (0.07)	0.22 ** (0.07)	0.12 (0.07)	0.20 ** (0.08)
**Unity leader**	0.34 *** (0.07)	0.40 *** (0.07)	0.22 ** (0.08)	0.25 ** (0.08)
Emphasizing the common goal	0.29 *** (0.07)	0.40 *** (0.07)	0.22 ** (0.07)	0.24 ** (0.08)
Putting the team interests before own interests	0.29 *** (0.07)	0.24 ** (0.07)	0.16 * (0.07)	0.13 (0.08)

Note. * *p* < 0.05; ** *p* < 0.01; *** *p* < 0.001.

**Table 5:** NCA effect sizes.

Leadership Roles and Their Underlying Functions (Defined by Edelmann et al. [12])	Team Effectiveness		OCB		Job Satisfaction		Team Cohesion	
	CE-FDH	*p*	CE-FDH	*p*	CE-FDH	*p*	CE-FDH	*p*
**Task leader**	0.21	0.05	0.23	0.03	0.10	0.60	0.10	0.43
Ensuring a fair division of tasks	0.13	0.06	0.19	0.01	0.01	0.43	0.05	0.30
Monitoring the work	0.14	0.08	0.18	0.05	0.11	0.04	0.04	0.65
Representing the team internally	0.18	0.03	0.21	0.01	0.12	0.20	0.07	0.52
Creating clarity about each other’s work	0.20	0.01	0.20	0.02	0.08	0.54	0.08	0.36
Coordinating tasks at team meetings	0.06	0.55	0.10	0.26	0.00	1.00	0.02	0.35
**Connecting leader**	0.10	0.71	0.16	0.18	0.07	0.65	0.10	0.37
Being a confidential advisor for colleagues	0.13	0.32	0.22	0.02	0.13	0.09	0.12	0.07
Cultivating a healthy work culture	0.13	0.14	0.23	0.00	0.06	0.43	0.10	0.07
Reducing tensions within the team	0.09	0.28	0.10	0.18	0.01	0.73	0.05	0.20
Opening up to colleagues	0.11	0.23	0.17	0.04	0.06	0.22	0.04	0.48
Ensuring connection and integration within the team	0.11	0.59	0.22	0.02	0.08	0.50	0.10	0.31
**Social activity leader**	0.08	0.67	0.19	0.00	0.06	0.32	0.01	0.72
Cultivating a good team atmosphere at work through social activities during work hours	0.08	0.45	0.15	0.04	0.05	0.11	0.01	0.80
Organizing social activities outside work hours	0.01	0.85	0.08	0.14	0.00	1.00	0.00	10.00
Paying attention to special events of team members	0.09	0.37	0.17	0.03	0.00	1.00	0.03	0.47
**Motivational leader**	0.24	0.05	0.31	0.02	0.23	0.16	0.16	0.23
Motivating colleagues to work	0.15	0.04	0.15	0.20	0.11	0.06	0.05	0.52
Encouraging colleagues to take initiative and voice their opinion	0.22	0.09	0.28	0.05	0.19	0.25	0.15	0.18
Recognizing the work of colleagues	0.24	0.02	0.29	0.02	0.19	0.18	0.17	0.14
Expressing gratitude	0.19	0.07	0.19	0.13	0.16	0.17	0.08	0.51
**Critical innovation leader**	0.14	0.04	0.21	0.00	0.06	0.32	0.04	0.46
Daring to take a critical look and initiating change	0.20	0.01	0.25	0.00	0.09	0.36	0.10	0.20
Daring to take initiative	0.10	0.62	0.18	0.17	0.00	1.00	0.10	0.15
Daring to criticize the formal leader	0.13	0.12	0.18	0.04	0.03	0.63	0.06	0.30
Preparing for the future	0.14	0.02	0.21	0.00	0.05	0.25	0.04	0.41
**Team-development leader**	0.17	0.08	0.26	0.00	0.11	0.44	0.09	0.32
Developing the growth and expertise of colleagues	0.14	0.05	0.20	0.04	0.10	0.18	0.04	0.70
Stimulating knowledge sharing	0.13	0.39	0.22	0.07	0.04	0.59	0.12	0.08
Giving colleagues feedback	0.14	0.05	0.24	0.00	0.07	0.09	0.05	0.36
Not wanting to do everything yourself	0.13	0.05	0.17	0.02	0.10	0.03	0.06	0.19
**External leader**	0.11	0.28	0.19	0.00	0.04	0.54	0.07	0.20
Representing the team to the formal leader	0.13	0.25	0.23	0.00	0.12	0.12	0.06	0.64
Representing the team externally	0.13	0.17	0.11	0.29	0.03	0.40	0.11	0.04
Connecting with external teams	0.05	0.71	0.15	0.01	0.00	1.00	0.00	10.00
**Logistics leader**	0.10	0.75	0.18	0.36	0.06	0.80	0.03	0.89
Paying attention to the logistics	0.08	0.62	0.17	0.09	0.00	1.00	0.02	0.74
Internal housekeeping	0.09	0.07	0.10	0.24	0.01	0.26	0.03	0.27
Paying attention to work safety	0.11	0.42	0.19	0.11	0.03	0.67	0.04	0.64
**Exemplary leader**	0.16	0.04	0.13	0.49	0.11	0.16	0.10	0.24
Being vulnerable	0.13	0.02	0.16	0.01	0.09	0.03	0.07	0.17
Acting as a role model	0.13	0.13	0.13	0.33	0.10	0.06	0.06	0.42
Giving feedback in a calm, polite manner	0.06	0.74	0.00	1.00	0.00	1.00	0.00	10.00
**Unity leader**	0.17	0.07	0.23	0.01	0.12	0.30	0.10	0.48
Emphasizing the common goal	0.11	0.40	0.16	0.14	0.08	0.21	0.01	0.89
Putting the team interests before own interests	0.17	0.05	0.20	0.07	0.13	0.19	0.10	0.10

**Table 6:** Results of the two-way interaction between each peer leadership role identified by Edelmann et al. [12] and the respective moderator variable for each of the four outcomes.

Leadership Role (Identified by Edelmann et al. [12])	Moderator	Team Effectiveness *β* (*SE*)	OCB *β* (*SE*)	Job Satisfaction *β* (*SE*)	Team Cohesion *β* (*SE*)
Task leadership	Team size	0.00 (0.10)	−0.06 (0.10)	0.16 (0.11)	0.07 (0.11)
	Team tenure	−0.02 (0.08)	0.08 (0.08)	0.11 (0.09)	0.05 (0.11)
	Team identification	−0.00 (0.07)	0.00 (0.07)	0.02 (0.08)	−0.02 (0.07)
Connecting leadership	Team size	0.04 (0.08)	0.04 (0.08)	0.15 (0.09)	0.08 (0.09)
	Team tenure	0.04 (0.06)	−0.04 (0.06)	−0.02 (0.07)	0.03 (0.07)
	Team identification	−0.09 (0.08)	−0.10 (0.08)	−0.05 (0.08)	−0.15 (0.08)
Social activity leadership	Team size	0.11 (0.09)	0.10 (0.09)	0.10 (0.09)	0.06 (0.10)
	Team tenure	−0.07 (0.07)	−0.05 (0.07)	−0.06 (0.08)	−0.13 (0.08)
	Team identification	−0.21 (0.08)	−0.00 (0.07)	−0.10 (0.08)	−0.12 (0.07)
Motivational leadership	Team size	0.02 (0.08)	−0.02 (0.09)	0.12 (0.10)	−0.06 (0.10)
	Team tenure	−0.04 (0.06)	−0.06 (0.07)	−0.02 (0.07)	−0.05 (0.07)
	Team identification	−0.03 (0.06)	0.02 (0.06)	−0.05 (0.06)	0.02 (0.06)
Critical innovation leadership	Team size	0.04 (0.08)	0.04 (0.08)	0.15 (0.09)	0.08 (0.09)
	Team tenure	0.04 (0.06)	−0.04 (0.06)	−0.02 (0.07)	0.03 (0.07)
	Team identification	−0.09 (0.08)	−0.10 (0.08)	−0.05 (0.08)	−0.00 (0.08)
Team-development leadership	Team size	−0.08 (0.08)	−0.13 (0.08)	0.08 (0.09)	−0.13 (0.10)
	Team tenure	−0.08 (0.07)	−0.05 (0.07)	−0.04 (0.07)	−0.06 (0.08)
	Team identification	−0.00 (0.06)	0.08 (0.06)	−0.01 (0.06)	0.06 (0.06)
External leadership	Team size	−0.04 (0.10)	−0.18 (0.10)	0.04 (0.11)	−0.09 (0.11)
	Team tenure	−0.24 (0.08) **	−0.09 (0.08)	−0.09 (0.09)	−0.11 (0.10)
	Team identification	−0.09 (0.06)	0.02 (0.06)	0.03 (0.06)	0.00 (0.06)
Logistics leadership	Team size	0.14 (0.08)	−0.01 (0.09)	0.07 (0.10)	0.04 (0.09)
	Team tenure	0.12 (0.09)	0.06 (0.10)	−0.06 (0.10)	0.03 (0.11)
	Team identification	−0.06 (0.07)	0.00 (0.07)	0.07 (0.07)	0.05 (0.07)
Exemplary leadership	Team size	0.02 (0.09)	0.01 (0.09)	0.16 (0.10)	−0.03 (0.10)
	Team tenure	0.00 (0.06)	−0.04 (0.06)	−0.01 (0.07)	0.00 (0.07)
	Team identification	−0.12 (0.06)	−0.01 (0.06)	−0.04 (0.06)	−0.01 (0.06)
Unity leadership	Team size	0.07 (0.08)	0.08 (0.09)	0.27 (0.09) ***	0.20 (0.11)
	Team tenure	−0.03 (0.07)	−0.04 (0.07)	−0.07 (0.08)	−0.03 (0.08)
	Team identification	−0.14 (0.06) **	−0.16 (0.06) *	−0.10 (0.06)	−0.26 (0.06) **

Note. * *p* < 0.05; ** *p* < 0.01; *** *p* < 0.005.

**Figure A1:**
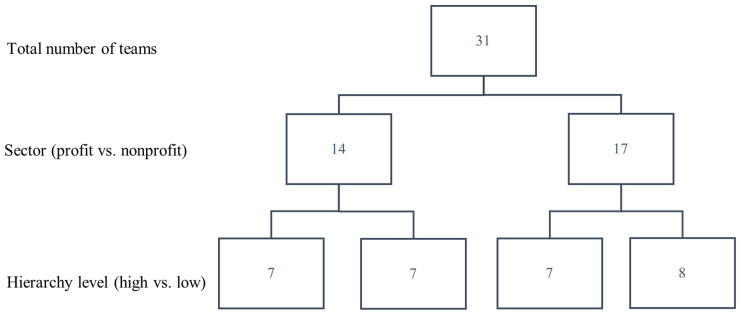
Participating Teams Stratified Across Sector and Hierarchy Levels.

**Figure A2:**
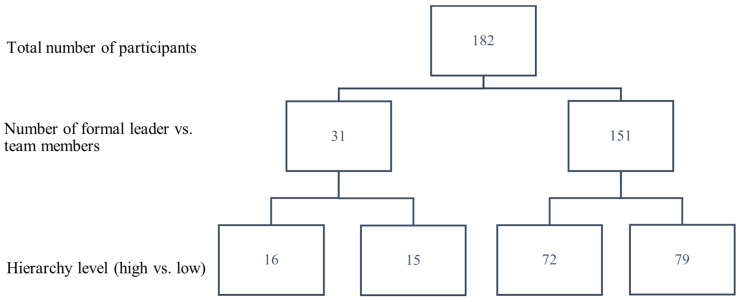
Individual Participants Stratified Across their Leadership Status and Hierarchy Level.

## Data Availability

The data presented in this study are available on request from the corresponding author. The data are not publicly available due to privacy reasons.

## Appendix C. Additional Analyses to Compare the Relevance of Roles in Profit vs. Non-Profit Organizations

In addition to the multilevel regression analyses conducted to address Aim 1, the comparison between profit and non-profit organizations emerged as an intriguing avenue for exploration, due to the possibility that not all leadership roles hold equal relevance within both organizational contexts. Evidently, the distinct nature of non-profit organizations, characterized by factors such as restricted resources and compensation, reliance on charitable contributions, and a mission-driven focus rather than profit-driven motives [98], suggests the potential need for a distinct leadership approach. Therefore, the same analyses for Aim 1 were performed separately for both types of organizations.

In general, the results indicate that the 10 peer leadership roles significantly contributed to positive outcomes within both profit and non-profit organizations. This observation underscores the relevance of these roles across diverse settings. In both types of organizations, each role predicted at least two outcomes, although a few exceptions were evident. Specifically, *External leadership* solely predicted OCB in profit organizations, while *Logistics leadership* only predicted team effectiveness in non-profit organizations. Below, we will briefly outline the three primary differences discerned between the two settings.

First, it is noteworthy that the roles of *Motivational* and *Exemplary leadership* both were more relevant within profit organizations (i.e., they contributed to all four outcomes) compared to their relevance within non-profit organizations. Second, the role of *Social activity leader* appeared to be more relevant within non-profit organizations compared to profit organizations. Third, both *Task* and *Critical innovation leadership* emerged as necessary conditions for most outcomes within profit organizations, while in non-profit organizations, this was only the case for one outcome, specifically OCB.

## Appendix D. Additional Analyses to Compare the Relevance of Roles in Teams with Low and High Levels of Power Distribution

Akin to the comparative analysis of different organizational types, we additionally explored whether the relations between the different roles and the four outcomes exhibited differences contingent upon team members’ perceptions of power distribution within their respective teams. To gauge the nature of power distribution within the team, we employed the “Authority differentiation” subscale derived from the Team Descriptive Index (TDI) by Lee et al. [99]. Participants were prompted to indicate the extent to which decision-making responsibilities (e.g., in the event of disagreements) were concentrated within an individual or diffused across the entire team, using a rating scale that ranged from 1 (*Our team has a strong leader in relation to other team members*) to 9 (*In our team*, *team members have an equal amount of power*). Accordingly, lower scores on this scale denoted teams wherein a more vertical leadership structure was perceived, characterized by centralized leadership vested primarily in a solitary individual. Conversely, higher scores indicated a perception of a more decentralized leadership structure, suggesting that power (i.e., leadership influence) was dispersed across the team.

Within our sample, an intermediate degree of power distribution was ascertained, with an average score of 5.82 (*SD* = 1.88). Subsequently, we conducted a correlation analysis to examine the relation between the 10 peer leadership roles and the four outcomes for either a high level (i.e., ≥5.82, *n* = 107) or a low level (i.e., <5.82, *n* = 75) of power distribution. Overall, the analyses yielded similar results. Across teams with both high and low power distribution, each role related significantly to at least one outcome, with the exception of the *Logistics leader* role, which exhibited no such correlations in either type of team. Moreover, the role of *Team-development leader* was related to all four outcomes, but exclusively within teams characterized by higher levels of power distribution. One possible explanation for this finding is that the impact of this role is less pronounced in teams that are inherently centralized, as these teams are less concerned with the development of fellow team members. Hence, regardless of the degree of power distribution prevailing within the team, 8 out of the 10 leadership roles retain their importance (e.g., the roles of *Task leader* and *Critical Innovation leader* related to team effectiveness and OCB in both types of teams).

Nonetheless, the findings also unveiled several differences between teams characterized by high versus low levels of power distribution. First, it is notable that within teams where employees perceive a lower degree of power distribution, the roles of *Connecting leader*, *Social activity leader*, and *External leader* only related to OCB. Conversely, within teams characterized by higher levels of power distribution, these same roles are also related to team effectiveness and job satisfaction. Second, the role of the *Motivational leader* seemed to be linked to team cohesion in teams with lower levels of power distribution. However, in teams with higher levels of power distribution, this role was associated with team effectiveness and OCB. Third, *Unity leadership* related to team effectiveness and OCB in teams with higher levels of power distribution, while in teams with lower power distribution, this role additionally correlated with job satisfaction and team cohesion. A plausible explanation for this finding lies in the possibility that in teams with a more centralized structure, the interconnections among team members may be relatively constrained compared to teams where leadership is shared among multiple members. As such, an individual within the team who undertakes the task of fostering unity and integration among team members could hold particular significance in teams characterized by lower power distribution.

## Appendix E

**Table A1:** CE–FDH Bottleneck Table.

	Percentage	Bottleneck Team Effectiveness	Bottleneck OCB	Bottleneck Job Satisfaction	Bottleneck Team Cohesion
Task leader	00	NN	NN	-	-
	10	NN	NN	-	-
	20	NN	NN	-	-
	30	NN	NN	-	-
	40	NN	NN	-	-
	50	NN	NN	-	-
	60	3.60	3.80	-	-
	70	3.60	4.20	-	-
	80	4.20	6.00	-	-
	90	6.20	8.60	-	-
	100	NA	NA	-	-
Connecting leader	00	-	-	-	-
	10	-	-	-	-
	20	-	-	-	-
	30	-	-	-	-
	40	-	-	-	-
	50	-	-	-	-
	60	-	-	-	-
	70	-	-	-	-
	80	-	-	-	-
	90	-	-	-	-
	100	-	-	-	-
Social activity leader	00	-	NN	-	-
	10	-	NN	-	-
	20	-	NN	-	-
	30	-	NN	-	-
	40	-	NN	-	-
	50	-	2.00	-	-
	60	-	2.67	-	-
	70	-	4.00	-	-
	80	-	4.67	-	-
	90	-	8.67	-	-
	100	-	NA	-	-
Motivational leader	00	NN	NN	-	-
	10	NN	NN	-	-
	20	NN	NN	-	-
	30	NN	NN	-	-
	40	NN	2.50	-	-
	50	NN	4.00	-	-
	60	4.00	4.00	-	-
	70	4.25	4.25	-	-
	80	5.00	8.00	-	-
	90	7.00	8.25	-	-
	100	8.00	NA	-	-
Critical innovation leader	00	NN	NN	-	-
	10	NN	NN	-	-
	20	NN	NN	-	-
	30	NN	NN	-	-
	40	NN	NN	-	-
	50	NN	NN	-	-
	60	NN	4.25	-	-
	70	NN	5.50	-	-
	80	4.50	6.50	-	-
	90	7.25	7.25	-	-
	100	8.75	NA	-	-
Team-development leader	00	-	NN	-	-
	10	-	NN	-	-
	20	-	NN	-	-
	30	-	NN	-	-
	40	-	2.50	-	-
	50	-	2.50	-	-
	60	-	4.00	-	-
	70	-	4.50	-	-
	80	-	6.75	-	-
	90	-	7.50	-	-
	100	-	NA	-	-
External leader	00	-	NN	-	-
	10	-	NN	-	-
	20	-	NN	-	-
	30	-	NN	-	-
	40	-	NN	-	-
	50	-	NN	-	-
	60	-	4.33	-	-
	70	-	4.33	-	-
	80	-	4.67	-	-
	90	-	8.33	-	-
	100	-	NA	-	-
Logistics leader	00	-	-	-	-
	10	-	-	-	-
	20	-	-	-	-
	30	-	-	-	-
	40	-	-	-	-
	50	-	-	-	-
	60	-	-	-	-
	70	-	-	-	-
	80	-	-	-	-
	90	-	-	-	-
	100	-	-	-	-
Exemplary leader	00	NN	-	-	-
	10	NN	-	-	-
	20	NN	-	-	-
	30	NN	-	-	-
	40	NN	-	-	-
	50	NN	-	-	-
	60	3.33	-	-	-
	70	3.33	-	-	-
	80	3.67	-	-	-
	90	4.33	-	-	-
	100	8.67	-	-	-
Unity leader	00	-	NN	-	-
	10	-	NN	-	-
	20	-	NN	-	-
	30	-	NN	-	-
	40	-	2.50	-	-
	50	-	2.50	-	-
	60	-	3.50	-	-
	70	-	3.50	-	-
	80	-	7.00	-	-
	90	-	7.00	-	-
	100	-	NA	-	-

Note. NN = not necessary; NA = not applicable. Empty cells, represented by a dash (“-”), indicate the absence of a significant necessary condition linking the leadership role with the respective outcome variable. Despite employing Likert scales for measurement, the constructs were treated as latent variable scores during the course of data analysis. To aid the interpretation of these scores, we present a percentile-based bottleneck table for the dependent constructs, which were partially assessed using diverse Likert scales. Conversely, the independent constructs (i.e., the leadership roles) were reconverted into their actual values, measured on a 10-point scale. To illustrate, the highest level (i.e., 100%) of team effectiveness can solely be achieved through an average Exemplary leadership score of 8.67.

## References

[1] van Dick R. (2023). Primary challenges for employee health and wellbeing [Perspective]. Front. Organ. Psychol..

[2] Kobal Grum D., Babnik K. (2022). The psychological concept of social sustainability in the workplace from the perspective of sustainable goals: A systematic review. Front. Psychol..

[3] Schönborn G., Berlin C., Pinzone M., Hanisch C., Georgoulias K., Lanz M. (2019). Why social sustainability counts: The impact of corporate social sustainability culture on financial success. Sustain. Prod. Consum..

[4] Macke J., Genari D. (2019). Systematic literature review on sustainable human resource management. J. Clean. Prod..

[5] Guest D.E. (2017). Human resource management and employee well-being: Towards a new analytic framework. Hum. Resour. Manag. J..

[6] de Jonge J., Peeters M.C.W. (2019). The vital worker: Towards sustainable performance at work. Int. J. Environ. Res. Public Health.

[7] Zhu J.L., Liao Z.Y., Yam K.C., Johnson R.E. (2018). Shared leadership: A state-of-the-art review and future research agenda. J. Organ. Behav..

[8] Nicolaides V.C., LaPort K.A., Chen T.R., Tomassetti A.J., Weis E.J., Zaccaro S.J., Cortina J.M. (2014). The shared leadership of teams: A meta-analysis of proximal, distal, and moderating relationships. Leadersh. Q..

[9] Pearce C.L., Conger J.A. (2003). Shared Leadership: Reframing the Hows and Whys of Leadership.

[10] Petermann MK H., Zacher H. (2021). Development of a behavioral taxonomy of agility in the workplace. Int. J. Manag. Proj. Bus..

[11] DeRue D.S., Morgeson F.P. Developing a taxonomy of team leadership behavior in self-managing teams. Proceedings of the 20th Annual Conference of the Society for Industrial and Organizational Psychology.

[12] Edelmann C., Boen F., Stouten J., Vande Broek G., Fransen K. (2023). Shared Leadership in Organisations: A Mixed-Method Approach. Doctoral Thesis.

[13] Drescher G., Garbers Y. (2016). Shared leadership and commonality: A policy-capturing study. Leadersh. Q..

[14] Wang L., Jiang W., Liu Z., Ma X. (2017). Shared leadership and team effectiveness: The examination of LMX differentiation and servant leadership on the emergence and consequences of shared leadership. Hum. Perform..

[15] Wang D., Waldman D.A., Zhang Z. (2014). A meta-analysis of shared leadership and team effectiveness. J. Appl. Psychol..

[16] Bergman J.Z., Rentsch J.R., Small E.E., Davenport S.W., Bergman S.M. (2012). The shared leadership process in decision-making teams. J. Soc. Psychol..

[17] Pearce C.L., Sims H.P. (2002). Vertical versus shared leadership as predictors of the effectiveness of change management teams: An examination of aversive, directive, transactional, transformational, and empowering leader behaviors. Group Dyn.-Theory Res. Pract..

[18] Hatami A., Hermes J., Keränen A., Ulkuniemi P. (2023). Creating social sustainability through distributing leadership and co-responsibility in corporate volunteering. South Asian J. Bus. Manag. Cases.

[19] Pearce C., Manz C., Akanno S. (2013). Searching for the holy grail of management development and sustainability: Is shared leadership development the answer?. J. Manag. Dev..

[20] Fausing M.S., Jeppe Jeppesen H., Jønsson T.S., Lewandowski J., Bligh M.C. (2013). Moderators of shared leadership: Work function and team autonomy. Team Perform. Manag. Int. J..

[21] Boies K., Lvina E., Martens M.L. (2010). Shared leadership and team performance in a business strategy simulation. J. Pers. Psychol..

[22] Mumford T., Van Iddekinge C., Morgeson F., Campion M. (2008). The team role test: Development and validation of a team role knowledge situational judgment test. J. Appl. Psychol..

[23] Yukl G., Mahsud R., Prussia G., Hassan S. (2019). Effectiveness of broad and specific leadership behaviors. Pers. Rev..

[24] Morgeson F.P., DeRue D.S., Karam E.P. (2010). Leadership in teams: A functional approach to understanding leadership structures and processes. J. Manag..

[25] D’Innocenzo L., Mathieu J.E., Kukenberger M.R. (2016). A meta-analysis of different forms of shared leadership–team performance relations. J. Manag..

[26] Fleishman E.A., Mumford M.D., Zaccaro S.J., Levin K.Y., Korotkin A.L., Hein M.B. (1991). Taxonomic efforts in the description of leader behavior: A synthesis and functional interpretation. Leadersh. Q..

[27] McGrath J. (1962). Leadership Behavior: Some Requirements for Leadership Training.

[28] Burke C.S., Stagl K.C., Klein C., Goodwin G.F., Salas E., Halpin S.M. (2006). What type of leadership behaviors are functional in teams? A meta-analysis. Leadersh. Q..

[29] Katz D., Kahn R.L. (1966). The Social Psychology of Organizations.

[30] Biddle B.J. (1979). Role Theory.

[31] Stewart G.L., Fulmer I.S., Barrick M.R. (2005). An exploration of member roles as a multilevel linking mechanism for individual traits and team outcomes. Pers. Psychol..

[32] Edelmann C.M., Boen F., Stouten J., Broek G.V., Fransen K. (2023). The advantages and disadvantages of different implementations of shared leadership in organizations: A qualitative study. Leadership.

[33] Burke C.S., Georganta E., Marlow S. (2019). A bottom up perspective to understanding the dynamics of team roles in mission critical teams. Front. Psychol..

[34] Driskell T., Driskell J., Burke S., Salas E. (2017). Team roles: A review and integration. Small Group Res..

[35] Mathieu J.E., Tannenbaum S.I., Kukenberger M.R., Donsbach J.S., Alliger G.M. (2014). Team role experience and orientation: A measure and tests of construct validity. Group Organ. Manag..

[36] Edelmann C.M., Boen F., Fransen K. (2020). The power of empowerment: Predictors and benefits of shared leadership in organizations. Front. Psychol..

[37] Fransen K., Vanbeselaere N., De Cuyper B., Vande Broek G., Boen F. (2014). The myth of the team captain as principal leader: Extending the athlete leadership classification within sport teams. J. Sports Sci..

[38] Manheim N. (2017). Shared Leadership in Teams: A Theoretical and Empirical Investigation. Ph.D. Thesis.

[39] Vanhove A.J., Herian M.N. (2015). Team cohesion and individual well-being: A conceptual analysis and relational framework. Team Cohesion: Advances in Psychological Theory, Methods and Practice.

[40] Faragher E.B., Cass M., Cooper C.L. (2005). The relationship between job satisfaction and health: A meta-analysis. Occup. Environ. Med..

[41] Wang W., Atingabili S., Mensah I.A., Jiang H., Zhang H., Omari-Sasu A.Y., Tackie E.A. (2022). Teamwork quality and health workers burnout nexus: A new insight from canonical correlation analysis. Hum. Resour. Health.

[42] Wang D., Ma E., Kim Y.S., Liu A., Berbekova A. (2021). From good soldiers to happy employees: Exploring the emotional and well-being outcomes of organizational citizenship behavior. J. Hosp. Tour. Manag..

[43] Dul J. (2016). Necessary Condition Analysis (NCA): Logic and Methodology of “Necessary but Not Sufficient” Causality. Organ. Res. Methods.

[44] Duchek S., Raetze S., Scheuch I. (2020). The role of diversity in organizational resilience: A theoretical framework. Bus. Res..

[45] Kozlowski SW J., Bell B.S. (2003). Work groups and teams in organizations. Handbook of Psychology: Industrial and Organizational Psychology.

[46] Cox J.F., Pearce C.L., Perry M.L., Pearce C.L., Conger J.A. (2003). Toward a model of shared leadership and distributed influence in the innovation process: How shared leadership can enhance new product development team dynamics and effectiveness. Shared Leadership: Reframing the Hows and Whys of Leadership.

[47] Curral L.A., Forrester R.H., Dawson J.F., West M.A. (2001). It’s what you do and the way that you do it: Team task, team size, and innovation-related group processes. Eur. J. Work Organ. Psychol..

[48] Wu Q., Cormican K., Chen G. (2018). A meta-analysis of shared leadership: Antecedents, consequences, and moderators. J. Leadersh. Organ. Stud..

[49] Wu Q., Cormican K. (2016). Shared leadership and team creativity: A Social Network Analysis in engineering design teams. J. Technol. Manag. Innov..

[50] Yukl G.A. (2010). Leadership in Organizations.

[51] Steffens N.K., Haslam S., Kerschreiter R., Schuh S., Dick R. (2014). Leaders enhance group members’ work engagement and reduce their burnout by crafting social identity. Ger. J. Hum. Resour. Manag..

[52] Kozlowski SW J., Ilgen D.R. (2006). Enhancing the effectiveness of work groups and teams. Psychol. Sci. Public Interest.

[53] Chen C.C., Wu J., Yang S.C., Tsou H.-Y. (2008). Importance of diversified leadership roles in improving team effectiveness in a virtual collaboration learning environment. J. Educ. Technol. Soc..

[54] Rafferty A.E., Jimmieson N.L. (2010). Team change climate: A group-level analysis of the relationships among change information and change participation, role stressors, and well-being. Eur. J. Work Organ. Psychol..

[55] Frenzel S.B., Junker N.M., Häusser J.A., Erkens V.A., van Dick R. (2023). Team identification relates to lower burnout—Emotional and instrumental support as two different social cure mechanisms. Br. J. Soc. Psychol..

[56] Kreft I., de Leeuw J. (1998). Introducing Multilevel Modeling.

[57] Welch C., Piekkari R. (2006). Crossing language boundaries: Qualitative interviewing in international business. Manag. Int. Rev..

[58] Podsakoff P.M., MacKenzie S.B., Lee J.Y., Podsakoff N.P. (2003). Common method biases in behavioral research: A critical review of the literature and recommended remedies. J. Appl. Psychol..

[59] Lee K., Allen N. (2002). Organizational citizenship behavior and workplace deviance: The role of affect and cognitions. J. Appl. Psychol..

[60] Hackman J.R., Oldham G.R. (1975). Development of the Job Diagnostic Survey. J. Appl. Psychol..

[61] Mathieu J.E. (1991). A cross-level nonrecursive model of the antecedents of organizational commitment and satisfaction. J. Appl. Psychol..

[62] Postmes T., Haslam S.A., Jans L. (2013). A single-item measure of social identification: Reliability, validity, and utility. Br. J. Soc. Psychol..

[63] R Core Team (2022). R: A Language and Environment for Statistical Computing.

[64] Scott-Young C.M., Georgy M., Grisinger A. (2019). Shared leadership in project teams: An integrative multi-level conceptual model and research agenda. Int. J. Proj. Manag..

[65] Hox J.J. (2002). Multilevel Analysis. Techniques and Applications.

[66] Dul J., Hauff S., Bouncken R.B. (2023). Necessary condition analysis (NCA): Review of research topics and guidelines for good practice. Rev. Manag. Sci..

[67] Costa S., Daher P., Neves P., Velez M.J. (2022). The interplay between ethical leadership and supervisor organizational embodiment on organizational identification and extra-role performance. Eur. J. Work Organ. Psychol..

[68] Wang L., Jiang M., Zhu F., Song P. (2022). Untangling employee well-being in projects: A configural analysis of job stressors and psychological needs. J. Manag. Eng..

[69] Goertz G., Starr H. (2002). Necessary Conditions: Theory, Methodology, and Applications.

[70] Dul J. (2023). Necessary Condition Analysis (NCA) and Its Diffusion.

[71] Dul J. (2020). Conducting Necessary Condition Analysis.

[72] Dawson J.F. (2014). Moderation in management research: What, why, when, and how. J. Bus. Psychol..

[73] Hulin C. (2001). Can a reliability coefficient be too high?. J. Consum. Psychol..

[74] Chiaburu D.S., Harrison D.A. (2008). Do peers make the place? Conceptual synthesis and meta-analysis of coworker effects on perceptions, attitudes, OCBs, and performance. J. Appl. Psychol..

[75] Bowers D.G., Seashore S.E. (1966). Predicting organizational effectiveness with a four-factor theory of leadership. Adm. Sci. Q..

[76] Humphrey S., Morgeson F., Mannor M. (2009). Developing a theory of the strategic core of teams: A role composition model of team performance. J. Appl. Psychol..

[77] Liang B., van Knippenberg D., Gu Q. (2021). A cross-level model of shared leadership, meaning, and individual creativity. J. Organ. Behav..

[78] Salanova M., Llorens S., Cifre E., Martínez I.M., Schaufeli W.B. (2003). Perceived collective efficacy, subjective well-being And task performance among electronic work groups: An experimental study. Small Group Res..

[79] Ogbonnaya C., Tillman C.J., Gonzalez K. (2018). Perceived organizational support in health care: The importance of teamwork and training for employee well-being and patient satisfaction. Group Organ. Manag..

[80] Wang X., Li C., Chen Y., Zheng C., Zhang F., Huang Y., Birch S. (2022). Relationships between job satisfaction, organizational commitment, burnout and job performance of healthcare professionals in a district-level health care system of Shenzhen, China. Front. Psychol..

[81] Gobet F. (2005). Chunking models of expertise: Implications for education. Appl. Cogn. Psychol..

[82] Carton A.M. (2022). The science of leadership: A theoretical model and research agenda. Annu. Rev. Organ. Psychol. Organ. Behav..

[83] Kerr S., Jermier J.M. (1978). Substitutes for leadership: Their meaning and measurement. Organ. Behav. Hum. Perform..

[84] Dietz B., van Knippenberg D., Hirst G., Restubog SL D. (2015). Outperforming whom? A multilevel study of performance-prove goal orientation, performance, and the moderating role of shared team identification. J. Appl. Psychol..

[85] Hoch J.E. (2007). Verteilte Führung in Virtuellen Teams: Zum Einfluss Struktureller, Interaktionaler und Teambasierter Führungstechniken auf den Teamerfolg [Distributed Leadership in Virtual Teams: The Impact of Structural, Interactive, and Team-Based Leadership on Team Success]. Ph.D. Thesis.

[86] To C., Yan T.T., Sherf E.N. (2021). Victorious and hierarchical: Past performance as a determinant of team hierarchical differentiation. Organ. Sci..

[87] Carson J.B., Tesluk P.E., Marrone J.A. (2007). Shared leadership in teams: An investigation of antecedent conditions and performance. Acad. Manag. J..

[88] Raudenbush S.W., Bryk A.S. (2002). Hierarchical Linear Models: Applications and Data Analysis Methods.

[89] Ragin C.C. (1987). The Comparative Method: Moving Beyond Qualitative and Quantitative Strategies.

[90] Mumford M.D., Scott G.M., Gaddis B., Strange J.M. (2002). Leading creative people: Orchestrating expertise and relationships. Leadersh. Q..

[91] Hanckel B., Petticrew M., Thomas J., Green J. (2021). The use of Qualitative Comparative Analysis (QCA) to address causality in complex systems: A systematic review of research on public health interventions. BMC Public Health.

[92] Crawford E.R., Lepine J.A. (2013). A configural theory of team processes: Accounting for the structure of taskwork and teamwork. Acad. Manag. Rev..

[93] Fransen K., Haslam S.A., Steffens N.K., Peters K., Mallett C.J., Mertens N., Boen F. (2020). All for us and us for all: Introducing the 5R Shared Leadership Program. Psychol. Sport Exerc..

[94] Friedrich T.L., Vessey W.B., Schuelke M.J., Ruark G.A., Mumford M.D. (2009). A framework for understanding collective leadership: The selective utilization of leader and team expertise within networks. Leadersh. Q..

[95] Contractor N.S., DeChurch L.A., Carson J., Carter D.R., Keegan B. (2012). The topology of collective leadership. Leadersh. Q..

[96] Pearce C.L. (2004). The future of leadership: Combining vertical and shared leadership to transform knowledge work. Acad. Manag. Perspect..

[97] United Nations: Sustainable Development Goals. https://www.un.org/sustainabledevelopment/sustainable-development-goals/.

[98] Allen S., Winston B.E., Tatone G.R., Crowson H.M. (2018). Exploring a model of servant leadership, empowerment, and commitment in nonprofit organizations. Nonprofit Manag. Leadersh..

[99] Lee S.M., Koopman J., Hollenbeck J.R., Wang L.C., Lanaj K. (2014). The Team Descriptive Index (TDI): A multidimensional scaling approach for team description. Acad. Manag. Discov..

